# Promotion of Nitrogen Fixation of Diverse Heterotrophs by Solid-Phase Humin

**DOI:** 10.3389/fmicb.2022.853411

**Published:** 2022-08-05

**Authors:** Sujan Dey, Takuya Kasai, Arata Katayama

**Affiliations:** ^1^Department of Civil and Environmental Engineering, Graduate School of Engineering, Nagoya University, Nagoya, Japan; ^2^Institute of Materials and Systems for Sustainability, Nagoya University, Nagoya, Japan

**Keywords:** humic substance, humin, biological nitrogen fixation, extracellular electron donation, diazotrophs, taxonomic diversity, enzymatic diversity

## Abstract

Although biological nitrogen fixation (BNF) proceeds under mild conditions compared to the energy-intensive Haber–Bosch process, the slow kinetics of BNF necessitate the promotion of BNF activity in its practical application. The BNF promotion using purified nitrogenases and using genetically modified microorganisms has been studied, but these enzymes are unstable and expensive; moreover, designing genetically modified microorganisms is also a difficult task. Alternatively, the BNF promotion in non-modified (wild-type) microorganisms (enriched consortia) with humin has been shown, which is a humic substance insoluble at any pH and functions as an extracellular electron mediator. However, the taxonomic distribution of the diazotrophs promoted by humin, the levels of BNF promotion, and the underlying mechanism in BNF promotion with humin remain unknown. In this study, we show that taxonomically diverse heterotrophic diazotrophs, harboring *nifH* clusters I, II, and III, promoted their BNF by accepting extracellular electrons from humin, based on the characterization of the individual responses of isolated diazotrophs to humin. The reduced humin increased the acetylene reduction activity of the diazotrophs by 194–916% compared to the level achieved by the organic carbon source, causing adenosine triphosphate (ATP) synthesis in the diazotroph cells without increase in the CO_2_ production and direct electron donation to the MoFe protein of the nitrogenase in the cells without relying on the biological electron transfer system. These would result in BNF promotion in the wild-type diazotroph cells beyond their biochemical capacity. This significant promotion of BNF with humin would serve as a potential basis for sustainable technology for greener nitrogen fixation.

## Introduction

Fixation of inert molecular nitrogen (N_2_) into a useable form, such as ammonia (NH_3_), is both energy-demanding and challenging (Cherkasov et al., 2015). The industrial Haber–Bosch (H–B) process is one of the main nitrogen fixation technologies, and close to 40% of the total world population depends on the nitrogen fertilizer generated by this process (Smil, 2001). However, the extreme energy requirement of this method (Cherkasov et al., 2015) makes this process environmentally unsustainable and thus urges to look for an alternative of the process. Biological nitrogen fixation (BNF) is a naturally occurring N-fixation process, on the other hand, it proceeds to fix further under ambient conditions. The BNF was firstly reported in 19th (in 1888) century in the root nodules of legumes in symbiotic association with *Rhizobia* (Burris and Roberts, 1993; Bazhenova and Shilov, 1995; Rascio and La Rocca, 2018). Later, BNF was also observed in various prokaryotes, including bacteria, cyanobacteria, and archaea known as free-living diazotrophs, as well as in many other symbiotic systems. The BNF in diazotrophs is carried out by the catalytic action of nitrogenases. All nitrogenases consist of two-protein cascade with a homodimeric reducing component called Fe protein (nitrogenase reductase) and a heterotetramer catalytic component (nitrogenase), which can be MoFe protein, VFe protein or FeFe protein (Hales et al., 1986; Müller et al., 1992). However, BNF is also energy demanding process, that is, requiring ATP hydrolysis, which is coupled with electron transfer between two protein components of nitrogenase (Eq. 1) (Alberty, 1994). Although metabolically expensive, BNF is a good process from an environmental viewpoint because it is eco-friendly, self-regulating, and uses renewable and environmentally agreeable substrates as energy sources. In addition, the diazotrophs are reluctant to fix N_2_ in the presence of other available nitrogen sources due to the high level of energy required (Burris and Roberts, 1993) as shown in Eq. 1.


(1) $$\begin{array}{l} \text{​​​​}{\text{N}}_{\text{2}}{\text{+8H}}^{\text{+}}{\text{+8e}}^{-}\text{ +16ATP}\to {\text{2NH}}_{\text{3}}{\text{+H}}_{\text{2}}\text{+16ADP+16Pi }\\ \text{ Δ}G^{{{}^{\circ}}^{\prime }}=-80.5\;KJ/mol \end{array}$$

Since the rate of BNF by diazotrophs is slow, the promotion of BNF is desired to widen its application in the practical field, as well as to decrease the dependency on chemical fertilizers for crop production (Chen et al., 2019). Therefore, there is a need to enhance the efficiency of BNF activity, as even a small improvement, when extrapolated to global-scale production, will substantially improve N-fixation output worldwide. The BNF promotion has been studied using purified nitrogenase through a direct electron transfer *via* an electron mediator (Brown et al., 2016; Milton et al., 2016, 2017; Milton and Minteer, 2017; Badalyan et al., 2019). However, the purified enzymes are expensive and show low conversion efficiency, poor substrate selectivity, and increased susceptibility to irreversible damage due to O_2_ exposure (Cherkasov et al., 2015; Ortiz-Medina et al., 2019). Recently, bioelectrochemical nitrogen fixation (e-BNF) has been reported to promote BNF activity using genetically engineered non-diazotrophic cyanobacteria with integrated nitrogenase gene clusters using extracellular electron supply through soluble electron mediator (Dong et al., 2021a). In e-BNF, the further-engineered cyanobacterium with transmembrane electron transfer ability has also been utilized in the absence of any extracellular electron mediator (Dong et al., 2021b). However, it is an arduous task to make such a genetically engineered microorganism because of the complicated and time-consuming procedure. Moreover, the use of soluble electron mediator (e.g., methyl viologen) has the following disadvantages: It is toxic to human and the environmental health, mediates contamination with the product, inefficient transmembrane diffusion, mediates inactivation, and low energy efficiency (Kamel, 2013; Dong et al., 2021b).

We recently showed the promotion of BNF activity of the wild-type microbial cells instead of genetically engineered microbial cells, for the first time, using anaerobic nitrogen-fixing consortia by supplying extracellular electrons through solid-phase electron mediator, humin (Dey et al., 2021). Humin is an organo–mineral humic substance, chemically stable and insoluble in any pH, eco-friendly, formed by decomposition of organic materials conditions, and has been reported to have quinone- and sulfur-containing redox-active moieties (Pham et al., 2021, 2022). Moreover, humin has been reported as an extracellular electron mediator to promote not only BNF but also various microbial reactions, including carbon dioxide fixation (Laskar et al., 2020), reductive dehalogenation (Zhang and Katayama, 2012; Laskar et al., 2019), denitrification (Xiao et al., 2016), and iron reduction (Zhang et al., 2015b). Undoubtedly, the promotion of BNF activity of anaerobic consortia through an extracellular electron supply is an important discovery in the field of e-BNF. The consortia with BNF promotion by humin were dominated by Clostridiales (Firmicutes), however, major *nifH* genes (nitrogenase reductases) in the consortia were not detected by specific PCR technique (Dey et al., 2021) in spite of using various degenerate primer sets for *nifH* genes (Gaby and Buckley, 2012). Therefore, the type of nitrogenase promoted by humin has remained unrevealed. The promotion effect of humin on the BNF activity of the wild-type diazotrophs other than Clostridiales also remains unknown. On top of that, the potential involvement of electroactive microorganisms in the extracellular electron transfer between humin and diazotrophs cannot be ruled out since the consortia were used. Based on these considerations, this study was conducted using isolates (type strains) of heterotrophic diazotrophs with diverse taxonomic positions (including Clostridiales) and different nitrogenases to understand comprehensively the BNF promotion of heterotrophic diazotrophs with humin. Examined were the direct utilization of extracellular electrons from solid-phase humin by heterotrophic diazotrophs, the taxonomic distribution and nitrogenase diversity of heterotrophic diazotrophs promoted by humin, and the promotion levels by humin in comparison with the ones achieved by substrates. The effects of a reduced humin on the ATP synthesis in the diazotroph cells and on the nitrogenase activity without nitrogenase reductase were also examined to reveal the interactions between humin and diazotroph cells.

## Materials and Methods

### Humin Preparation

Humin was extracted from the surface soil of the Kamajima paddy field (Endoaquept, Yatomi City, Aichi Prefecture, Japan) using a previously described method (Zhang et al., 2015a) with some modifications. In brief, the soil was air dried and sieved using a 1-mm mesh sieve. The sieved soil (100 g) was repeatedly washed by shaking with a chemical solution (24 h), followed by centrifugation (8,000 g, 15 min, 24°C) and decantation. Chemical solutions for washing were as follows: Ten times with 150 ml of 2% HF, 10 times with 150 ml of 0.1 M NaOH, 10 times with 150 ml of 2 % HF, and 20 times with 150 ml of ultrapure water. After washing, the pH was adjusted to 7.0, using 0.1-M NaOH. The pH-adjusted humin was freeze dried, ground using a ceramic mortar and pestle, and subjected to the experiments.

### Type Strains of Diazotrophs

Thirteen N-fixing bacterial type strains of different phyla and one N-fixing archaeon were used: *Azorhizobium caulinodans* JCM 20966, *Ensifer fredii* JCM 20967, *Rhodobacter sphaeroides* JCM 6121, *Pelomonas saccharophila* JCM 15912, *Derxia gummosa* JCM 20996, *Rubrivivax gelatinosus* JCM 21318, *Azotobacter vinelandii* JCM 21475, *Pseudomonas stutzeri* JCM 5965, *Geobacter sulfurreducens* DSMZ 12127, *Paenibacillus macerans* JCM 2500, *Clostridium pasteurianum* JCM 1408, *Clostridium tyrobutyricum* JCM 11008, *Nocardia cellulans* JCM 9965, and *Methanosarcina barkeri* JCM 10043. These strains were purchased from Japan Collection of Microorganisms (JCM) at the RIKEN BioResource Research Center (Tsukuba, Ibaraki, Japan), through the National Bio-resource Project of the Ministry of Education, Culture, Sports, Science, and Technology (MEXT), Japan, and from the DSMZ-German Collection of Microorganisms and Cell Cultures GmbH, Germany. They were maintained as cultures in the media recommended by JCM or DSMZ, as shown in Table 1.

**Table 1:** List of the studied diazotrophs with their culture conditions.

**Diazotroph name**	**Medium for maintenance**	**Organic carbon source used in 1 L of MNDA medium**	**Culture condition**
*A. caulinodans* JCM 20966	JCM 254^*^	20-g mannitol	30°C, Aerobic
*E. fredii* JCM 20967	JCM 254	20-g mannitol	30°C, Aerobic
*R. sphaeroides* JCM 6121	JCM 26	0.5-g glucose, 0.5-g soluble starch, 0.3-g Na-pyruvate	30°C, Anaerobic
*P. saccharophila* JCM 15912	JCM 346	0.5-g glucose, 0.5-g soluble starch, 0.3-g Na-pyruvate	30°C, Aerobic
*D. gummosa* JCM 20996	JCM 346	0.5-g glucose, 0.5-g soluble starch, 0.3-g Na-pyruvate	30°C, Micro -aerophilic
*R. gelatinosus* JCM 21318	JCM 358	10-g mannitol, 1-g Na-acetate, 1-g Na-succinate	30°C, Anaerobic
*A. vinelandii* JCM 21475	JCM 346	0.5-g glucose, 0.5-g soluble starch, 0.3-g Na-pyruvate	30°C, Aerobic
*P. stutzeri* JCM 5965	Nutrient broth with 0.5% NaCl	0.5-g glucose, 0.5-g soluble starch, 0.3-g Na-pyruvate	30°C, Aerobic
*G. sulfurreducens* DSMZ 12127	DSMZ 826^**^	1-g Na-acetate	35°C, Anaerobic
*P. macerans* JCM 2500	JCM 75	4-g glucose	30°C, Aerobic
*C. pasteurianum* JCM 1408	JCM 75	4-g glucose, 0.5-g soluble starch	37°C, Anaerobic
*C. tyrobutyricum* JCM 11008	JCM 75	4-g glucose, 0.5-g soluble starch	37°C, Anaerobic
*N. cellulans* JCM 9965	JCM 475	4-g glucose	30°C, Aerobic
*M. barkeri* JCM 10043	JCM 230	10-g mannitol, 10-ml methanol/L	37-°C, Anaerobic

* *JCM culture media: https://jcm.brc.riken/en/*.

** *DSMZ culture media: https://www.dsmz.de/collection/catalog/microorganims/culture-technology/list-of-media-for-microorganisms*.

### Culture Media for Examining N-Fixation

Three modified nitrogen-deficient Ashby (MNDA) media (Ashby, 1907) were used to examine the N-fixation ability of the individual-type strains. The anaerobic medium was composed of MgSO_4_·7H_2_O (0.2 g/L), K_2_SO_4_ (0.1 g/L), NaCl (0.2 g/L), K_2_HPO_4_ (0.2 g/L), CaCO_3_ (5 g/L), trace elements, vitamins, and an organic carbon source. The trace elements were provided by the addition of 1 ml/L of trace element solution SL-10 (Widdel et al., 1983) and vitamins by 10 ml/L vitamin solution (Sanford et al., 1996). The anaerobic condition of the medium was achieved by sparging with N_2_ gas for 90 min, followed by flushing the headspace for 5 min. The different organic carbon sources used for the individual strains along with the recommended incubation temperatures, are summarized in Table 1, along with the recommended incubation temperature. The anaerobic MNDA HEPES medium was obtained by replacing the CaCO_3_ buffer with 30-mM HEPES, and the anaerobic MNDA ${\text{HCO}}_{3}^{-}$ medium was prepared by replacing CaCO_3_ with NaHCO_3_ (0.85g/L) and N_2_ gas with a mixture of N_2_ and CO_2_ gases (80:20) for 90 min followed by flushing the headspace for 5 min.

### Preparation of Washed and Starved Culture

To examine the effect of humin on the N-fixation activity of the individual diazotrophs, washed and starved cultures were prepared. The maintained individual diazotrophs grown in the recommended medium were washed three times, suspended in organic carbon source-free MNDA HEPES medium, and by decanting the supernatant after centrifugation at 2,500 g for 5 min at 4°C. The washed individual microbial biomass was transferred into 20 ml of anaerobic MNDA HEPES medium with an organic carbon source in 50 ml-volume vial and incubated under anaerobic conditions at the recommended temperature (30, 35, or 37°C depending on the diazotrophs, as shown in Table 1) for 2 weeks. Subsequently, the cultured individual diazotrophs were rewashed three times to remove the residual organic carbon source. The washed culture was then transferred to the organic carbon source-free anaerobic MNDA HEPES medium and incubated for 2 weeks for starvation, after which the washed and starved individual cultures were subjected to further experiments.

### Preparation of Humin With Different Redox States

The effect of the different redox states of humin on the N-fixation activity of the type strains was examined. An oxidized and a reduced humin were prepared in 200 ml of 0.5-M Na_2_SO_4_ solution using an electrochemical system consisting of a potentiostat (Automatic Polarization System HSV-110, Hokuto Denko, Osaka, Japan), two twisted platinum electrodes (1 m in length and 0.8 mm in diameter) as the working and counter electrodes, and an Ag/AgCl reference electrode [+0.199 V *vs*. the standard hydrogen electrode (SHE)], under anaerobic conditions using a vinyl anaerobic chamber (Coy-7450000, COY, Grass Lake, MI, USA). Two grams of autoclaved humin were reduced or oxidized by maintaining the redox potential at −0.4 V or +0.4 V (*vs*. SHE) for 24 h, respectively. After reaching the potential at the equilibrium state, the reduced and oxidized humin were collected by filtration through filter paper, dried using a vacuum pump, and subjected to the experiments.

### Acetylene Reduction Activity (ARA) Assay

The ARA assay is a widely used method for examining N-fixation activity (Hardy et al., 1968, 1973). Therefore, an ARA assay was performed to assess the activity of the nitrogenase enzyme of the individual diazotrophs, which reduces N_2_ to NH_3_ and other substrates, including the reduction of acetylene to ethylene (Burris, 1991). The ARA assay was conducted using a 10 ml glass vial (referred to as the test vial). The test vial was autoclaved and sealed with a butyl rubber stopper and aluminum seal to ensure that no air exchange occurred. When the effect of humin was examined, 0.03 g (15 g/L) of humin (intact, oxidized, or reduced) was added anaerobically before sealing the test vial. The test vial was flushed with He gas aseptically to expel the air inside the vial, and 2 ml of the anaerobic culture medium (organic carbon source-free MNDA medium) was added. Then, 2 ml of washed and starved culture was aseptically transferred to each test vial, followed by a 200-μl acetylene gas injection. The ARA assays of two strains, *A. vinelandii* and *C. pasteurianum*, were also performed in the medium with vanadium as sodium orthovanadate dihydrate, Na_3_VO_4_·2H_2_O, by replacing Na_2_MoO_4_·2H_2_O at the same concentration in SL-10 solution (MNDA V medium). The amounts of acetylene and ethylene on day 0 (before incubation) and day 7 (after incubation) in the headspace were determined using a GC-14B gas chromatograph (Shimadzu, Kyoto, Japan) equipped with a Molecular Sieve-5A column (60/80 mesh, 3-mm inner diameter, and 2-m length), a Porapak N column (50/80 mesh, 3-mm inner diameter, and 3-m length) in a series, and with a thermal conductivity detector and a flame ionization detector, following the procedure described in a previous study (Dey et al., 2021).

### Determination of Total Kjeldahl Nitrogen (TKN) and H_2_ Production in the Cultures of the Individual Diazotrophs With Humin at Different Redox States

Nitrogen fixation activity of the individual diazotrophs was also determined by measuring the total Kjeldahl nitrogen using the Kjeldahl digestion method (Kjeldahl, 1883) followed by spectrophotometric measurement of ammonium ion using the indophenol blue method (Keeney and Nelson, 1982). Serum vials (50 ml volume) were prepared with 25 ml of organic carbon source-free anaerobic MNDA medium. The medium was supplemented with intact humin (15 g/L), oxidized humin (15 g/L), or the reduced humin (15 g/L) before the vials were sealed. Medium without humin was also provided. For *M. barkeri*, an organic carbon source-free MNDA ${\text{HCO}}_{3}^{-}$ medium was used. The medium was inoculated with 2.5 ml of washed and starved cultures of the individual diazotrophs, and incubated for 14 days at an appropriate temperature (30, 35, or 37°C depending on the diazotrophs; Table 1). Before and after 14 days of incubation, 5 ml of the culture sample was collected using a syringe and subjected to Kjeldahl nitrogen determination. In addition, H_2_ in the headspace was measured on day 0 (before incubation) and day 14 (after incubation) using a GC-14B gas chromatograph (Shimadzu, Kyoto, Japan) equipped with a thermal conductivity detector, as described above. The experiment was performed at least in triplicate for each type of strain.

### Determination of Respiration Rate and ARA

To examine whether humin works as a carbon source for promoting the N-fixation activity of the individual diazotrophs, the total CO_2_ was analyzed along with the ARA assay. The serum vials with 50-ml volume were filled with 35 ml of organic carbon source-free anaerobic MNDA HEPES medium in an anaerobic chamber and inoculated with the washed and starved culture 3.5 ml after the headspace was flushed with He gas for 5 min. One set of vials was supplemented with 15 g/L of the reduced humin and another set without humin. All vials were spiked with 1 ml of acetylene gas, and the prepared cultures were incubated for 14 days at an appropriate temperature (Table 1). Before and after the incubation, CO_2_, acetylene, and ethylene in the headspace were determined using a GC-14B gas chromatograph (Shimadzu, Kyoto, Japan) equipped with a thermal conductivity detector and a flame ionization detector (described above). Aqueous phase ${\text{HCO}}_{3}^{-}$ determination was performed by taking 15 ml of sample from the culture using a syringe before (0-day sample) and after incubation (14th-day sample). The samples were filtered using a membrane filter with 0.22-μm pore size (Millex^®^-Gv, Durapore™ PVDF membrane), and the ${\text{HCO}}_{3}^{-}$ concentration in the filtered sample was determined using a total organic carbon analyzer (TOC–VCPH, Shimadzu, Kyoto, Japan). For *A. vinelandii* and *C. pasteurianum*, the organic carbon source-free anaerobic MNDA HEPES V medium was also provided by replacing Na_2_MoO_4_·2H_2_O in the SL-10 solution with Na_3_VO_4_·2H_2_O at the same concentration. For other diazotrophs, Na_2_MoO_4_·2H_2_O containing the SL-10 solution was used.

### Determination of Carbon-Saturated Concentration of the Anaerobic MNDA Medium for Maximum ARA of the Diazotrophs

To determine the carbon saturation condition for maximum ARA of the individual diazotrophs, anaerobic MNDA medium containing different concentrations (up to 6 times of the standard concentration) of organic carbon was prepared. For the ARA assay, 2 ml of anaerobic MNDA medium with different organic carbon concentrations was added to six sets (with at least three replicates each) of test vials, and an ARA assay was performed as described above after the inoculation of 2 ml of washed and starved culture. The organic carbon concentration, defined as the organic carbon-saturated concentration at which diazotrophs exhibited the maximum ARA, was determined. The standard concentration of organic carbon (1 ×) was 0.125 g/L of glucose, 0.125 g/L of soluble starch, and 0.075 g/L of Na-pyruvate for *A. vinelandii*; 1 g/L of glucose and 0.125 g/L of soluble starch for *C. tyrobutyricum* and *C. pasteurianum*; for *E. fredii*, it consisted of 5 g/L of mannitol; and for *P. macerans*, it was 1 g/L glucose. For *A. vinelandii* and *C. pasteurianum*, the medium used was anaerobic MNDA V medium supplemented with Na_3_VO_4_·2H_2_O, replacing Na_2_MoO_4_·2H_2_O in SL-10 solution with the same concentration of Na_3_VO_4_·2H_2_O; these diazotrophs had vanadium nitrogenase. For other diazotrophs, an SL-10 solution containing Na_2_MoO_4_·2H_2_O was used for molybdenum nitrogenase.

### The ARA Promotion of the Diazotrophs by Reduced Humin Under the Carbon-Saturated Condition

The ARA assay of the individual diazotrophs was performed by incubation with the inoculation with washed and starved culture under carbon-saturated conditions with the reduced humin. The autoclaved test vials were provided, allowing five sets (with at least three repetitions each), and the first to fourth sets were supplemented with 15, 30, 45, and 60 g/L of the reduced humin, respectively; the fifth set contained no humin. The test vials were added aseptically with 2 ml of He-bubbled anaerobic MNDA medium with 3- or 4-times organic carbon concentration (saturated concentration), followed by 2 ml of the washed and starved culture inoculation. After injecting 200 μl of acetylene gas into each vial, an ARA assay was performed on day 0 (before incubation) and day 7 (after incubation). For *A. vinelandii* and *C. pasteurianum*, a He-bubbled anaerobic MNDA V medium was used. For other diazotrophs, a normal SL-10 solution containing Na_2_MoO_4_·2H_2_O was used.

### Total Bacterial Count (TBC) by Fluorescence Microscopy

The bacterial cultures were serially diluted (from 10^−1^ to 10^−4^) with PBSE buffer (130-mM NaCl, 1 mM EDTA, 30 mM K_2_HPO_4_, 30 mM KH_2_PO_4_, pH 7.0), filtered using a membrane filter with 0.2 μm pore size, and dried using a vacuum pump. Then, a drop of DAPI solution (ProLong^TM^ Gold, Thermo Fisher Scientific, Waltham, MA, USA 02451) was placed over the membrane filter, the samples were kept in the dark for 2 h, and observed under a fluorescent microscope (Olympus DP70, Tokyo, Japan) using an excitation filter WU (Blue) with an excitation wavelength of 320–385 nm.

### The ATP Assay

The ATP assay was performed using the CellTiter-Glo^®^ 2.0 assay (G9241, Promega Corporation, Madison, WI, USA) and Victor nivo™ multimode plate reader (PerkinElmer, Inc. Waltham, MA, USA). Serum vials (50 ml volume) were provided with 20 ml of carbon source-free MNDA HEPES medium, which was bubbled with N_2_ for 90 min, and the headspace was flushed with N_2_ for 5 min prior to inoculation. For *A. vinelandii* and *C. pasteurianum*, the medium used was an organic carbon source-free anaerobic MNDA HEPES V medium, and *M. barkeri* had an organic carbon source-free MNDA ${\text{HCO}}_{3}^{-}$ medium. One set of vials was supplemented with the 15-g/L reduced humin, and another set was not supplemented. Two milliliters of the washed and starved cultures were aseptically inoculated into each vial. Before incubation (day 0) and after incubation (day 3), an ATP assay was performed with 100 μl of culture according to the manufacturer's protocol. In brief, 100 μL of the sample was transferred to an opaque-walled multiwell plate, and an equal volume of CellTiter-Glo^®^ 2.0 was added to the sample, and the contents were mixed for 2 min using an orbital shaker. The plate was then incubated at 23°C for 10 min to stabilize the luminescent signal, and luminescence was recorded at 700 nm.

### Construction of Nitrogenase Overexpression Mutant

To construct the nitrogenase overexpression mutant, *nifDK* genes (gene ID: AOZ74834 and AOZ74835), from *C. pasteurianum* JCM 1408 (Supplementary Figure S1C) that encodes nitrogenase, were amplified using Phusion High-Fidelity DNA Polymerase (New England BioLabs, MA, USA), using *nifDK* primers *nifDK*-F_KpnI (5′- GGGG*GGTACC*TTTAATTTTGATGAGGGGTG−3′) and *nifDK*-R_XbaI (5′- GG*TCTAGA*TGTCAATTCCATATAGAATGAC−3′), where the italic sequences denote restriction sequences for cloning. The PCR product was purified using a QIAquick PCR Purification Kit (Qiagen, Venlo, Netherlands) according to the manufacturer's instructions. The purified PCR product and cloning vector pBBR1MCS-2 were digested with KpnI and XbaI (New England BioLabs, MA, USA), and the digested PCR product was cloned into the digested pBBR1MCS-2 (pBBR*nifDK*) (Supplementary Figure S1A). Also, pBBR*nifDK* was introduced into *Escherichia coli* JM109λ*pir*, derived from the *E. coli* K-12 strain. The expression of *nifD* and *nifK* in the constructed mutant was confirmed by qRT-PCR to be induced by 100-μM isopropyl β-D-1-thiogalactopyranoside (IPTG). The mutant was designated *nifDK*-OE.

### Total RNA Extraction

The *nifDK*-OE mutant was cultured in a 20-ml N-free M9 medium containing 100 μM or no IPTG at an initial optical density at 600 nm (OD_600_) of 0.5 under anaerobic conditions at 30°C, and harvested 1 h after incubation. The total RNA was extracted using ISOGEN (Nippon Gene Co., Tokyo, Japan) according to the manufacturer's protocol. The extracted RNA was purified using the RNeasy Mini Kit and RNase-free DNase set (Qiagen, Venlo, Netherlands). The concentration and quality of the purified RNA were evaluated using a DeNovix DS-11 spectrophotometer (Denovix Inc., DE, USA) and the Agilent 4150 TapeStation system with RNA ScreenTape and RNA ScreenTape reagents (Agilent Technologies Inc., CA, USA) according to the manufacturer's protocol.

### Quantitative Reverse Transcription PCR (qRT-PCR)

Quantitative reverse transcription PCR (qRT-PCR) was conducted using a LightCycler 1.5 instrument with LightCycler RNA Master SYBR Green I (Roche, Basel, Switzerland) following the manufacturer's protocol (Kouzuma et al., 2012). Briefly, a PCR reaction mixture contained 25-ng total RNA, 1.3 μl of 50-mM Mn(OAc)_2_ solution, 7.5-μl of LightCycler RNA Master SYBR Green I (Roche), and 0.15 μM primer sets [16S rRNA: 341F-518R (Luo et al., 2010), *nifD*: qrt-*nifD*-F 5′-GGTTGTGCTTATGCAGGATG-3′ and trt-*nifD*-R 5′-TCCTATAGGTCCGTGTGTGATG-3′, *nifK*: qrt-*nifK*-F 5′-GTTGCATTACTTGGAGATCCTG-3′ and qrt-*nifK*-R 5′-CTTCTGCAAGCATAGCATCG-3′) (Supplementary Figure S1I]. The DNA fragments of target genes (16S rRNA, *nifD*, and *nifK*) were amplified by PCR using Ex Taq polymerase (Takara Bio Inc., Shiga, Japan) and the same primer sets and purified by gel electrophoresis using a QIAEXII gel extraction kit (Qiagen, Venlo, Netherlands) according to the manufacturer's instructions to generate a standard curve. Standard curves were generated by amplifying a series of purified DNA fragments of each gene. Expression levels of target genes (*nifD* and *nifK*) were normalized based on the reference gene expression levels (16S rRNA).

### Operation of Electrochemical Cells

The *nifDK*-OE mutant was cultivated in a single-chambered electrochemical reactor (115 ml capacity, Supplementary Figure S1B) under anaerobic conditions. The reactor was filled with 80 ml of modified N-free M9 medium, in which NH_4_Cl was removed (LaCroix et al., 2015). The *nifDK*-OE cells were cultivated in N-free M9 medium at 30°C for 2 h to consume the nitrogen compounds in cells completely and were then inoculated into the medium containing 50 μg/ml kanamycin and 100-μM IPTG at an OD_600_ of 0.01. When necessary, 5 g/L of humin was added to the medium. The reactor was sealed with a cap equipped with a carbon rod (diameter, 5 mm; length, 15 cm) as the working electrode; platinum wire (diameter, 0.8 mm; length, 1 m) as the counter electrode; and Ag/AgCl as the reference electrode, and purged with pure nitrogen gas for 30 min. The working electrode was poised at −0.5 V *vs*. SHE using a potentiostat (Automatic Polarization System HSV-110, Hokuto Denko, Osaka, Japan).

### The DNA Extraction

For the extraction of DNA from the *nifDK*-OE mutant, *nifDK*-OE cells incubated in the electrochemical reactor were collected after 0 and 72 h of incubation and subjected to the extraction of total DNA using the FastDNA SPIN Kit for Soil (MP Biomedicals, CA, USA) according to the manufacturer's instructions. The purified DNA was dissolved in 50 μl of DES solution in the kit.

### Quantitative PCR (qPCR)

The abundance of the target genes (16S rRNA) in the cultures was measured by quantitative PCR. The standard DNA fragments were amplified using primer sets 341F-518R for 16S rRNA (Dey et al., 2021) and purified using the QIAquick PCR Purification Kit (Qiagen, Valencia, CA, USA). The qPCR was performed using LightCycler systems and a LightCycler DNA Master SYBR Green I (Roche Diagnostics, Basel, Switzerland), as previously described (Dey et al., 2021).

### Statistical Analysis

Statistical analysis was performed using *t*-test, two-tailed Pearson's correlation assignment, or one-way ANOVA followed by *post hoc* analysis by Tukey's method using the software IBM, SPSS, v.21.

## Results

### Acceleration of a Wide Range of Diazotrophs by Extracellular Electron Donation From Humin

**Figure 1:**
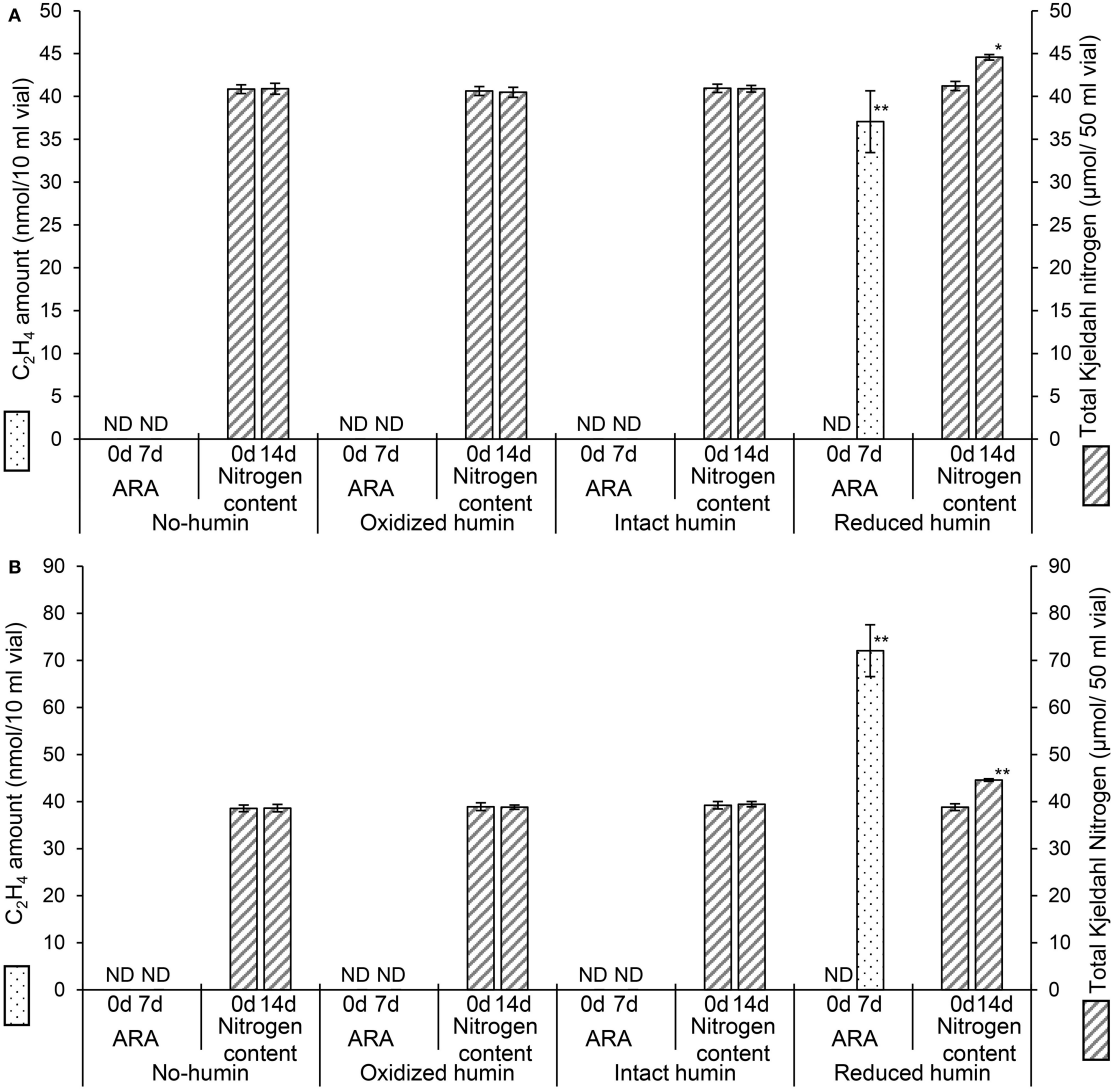
Changes in acetylene reducing activity (ARA), as the amount of ethylene produced, and total Kjeldahl nitrogen (TKN) of the washed and starved *C. pasteurianum* **(A)** and *A. vinelandii* **(B)** with different redox states of humin during 7 days (for ARA) and 14 days (for TKN) of incubation. No organic carbon source was added to the culture medium. Controls without inoculation showed no ARA and no increase in nitrogen content (data not shown). ND: not detected. Symbols “^*^” and “^**^” denote significant differences at *p* < 0.05, and *p* < 0.01, respectively.

**Figure 2:**
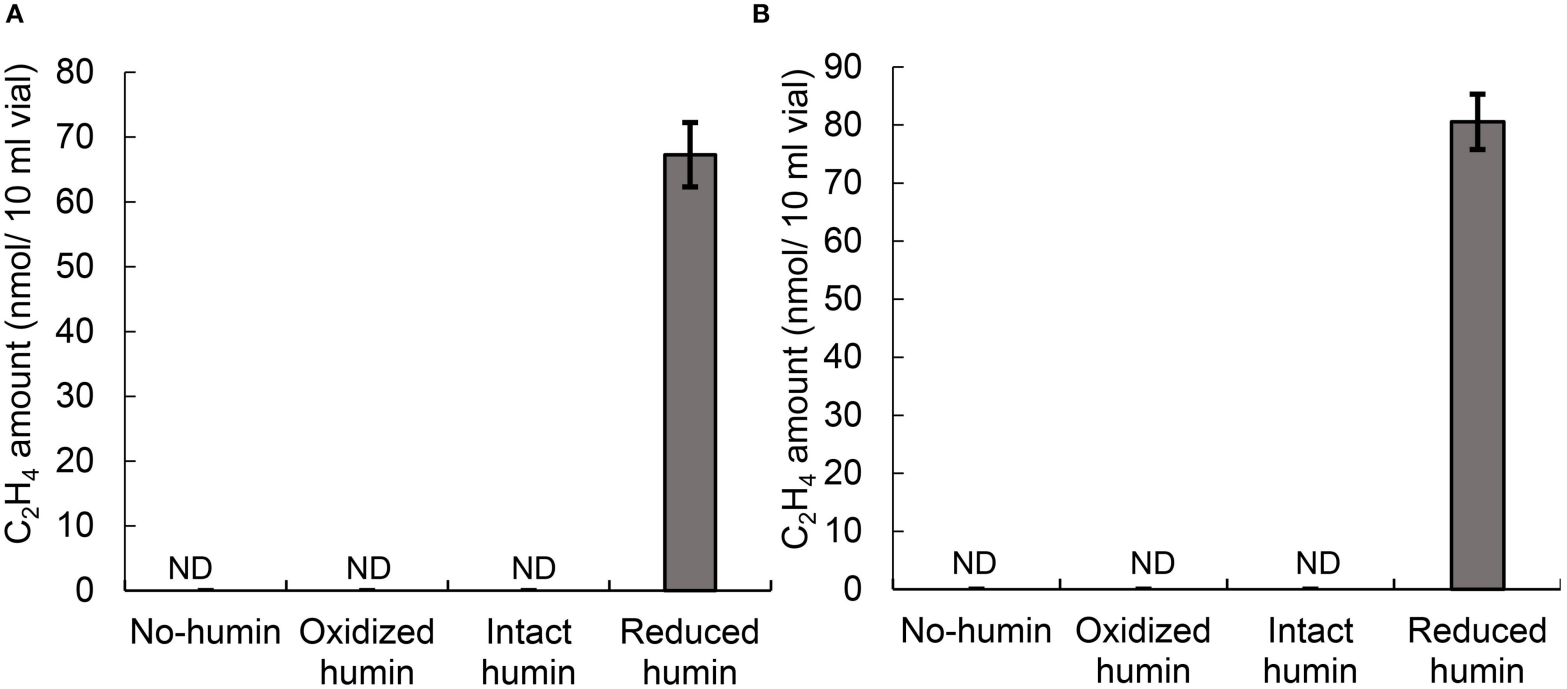
ARA, as the amount of ethylene produced, of *C. pasteurianum* **(A)** and *A. vinelandii* **(B)** with different redox states of humin under vanadium conditions after seven days of incubation. No organic carbon source was added to the culture medium. No ARA was detected on day 0 (data not shown). Controls without inoculation did not show any ARA on days 0 and 7 (data not shown). ND, not detected.

**Figure 3:**
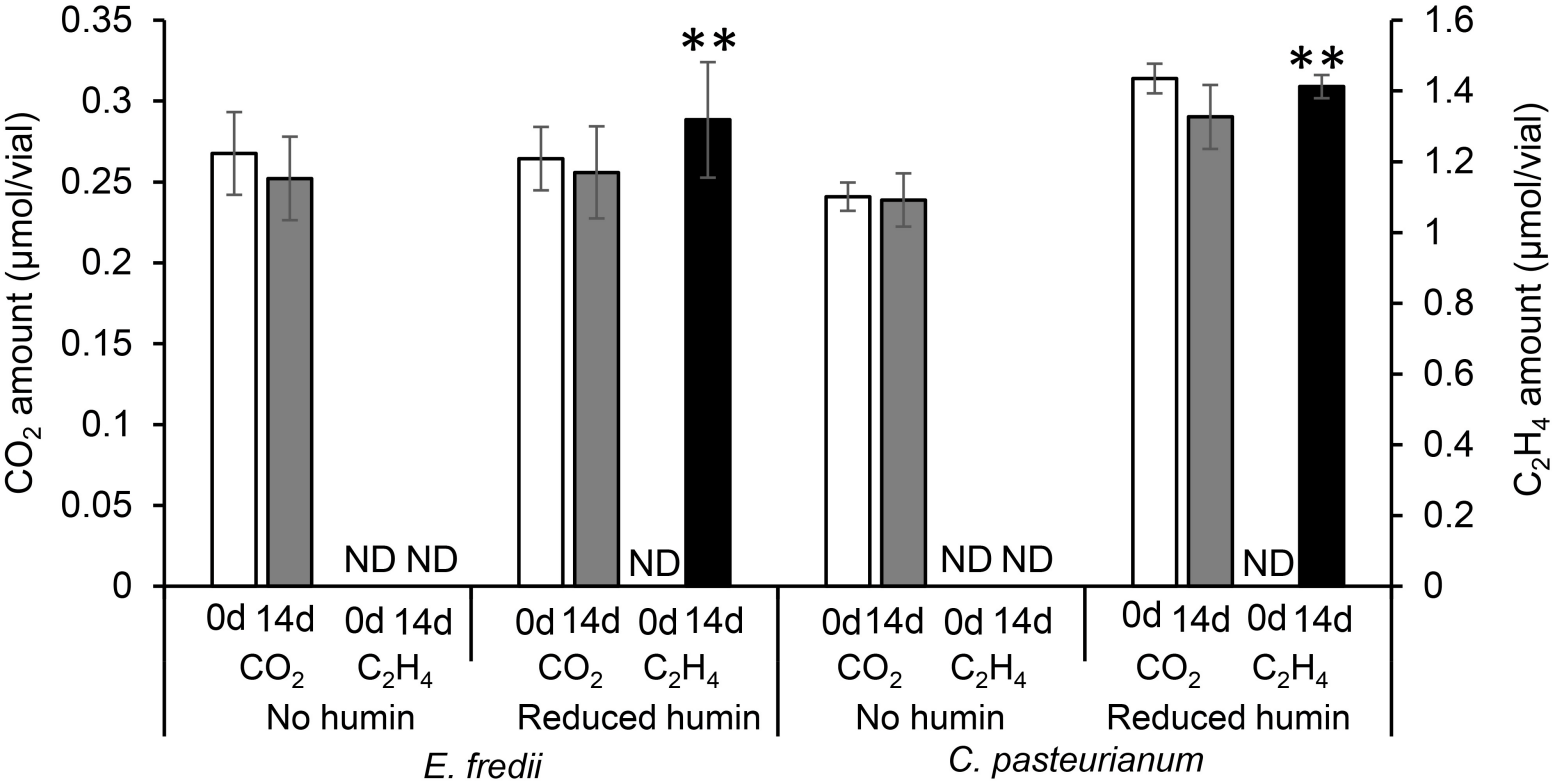
The CO_2_ amount and ARA, as the amount of ethylene produced, in the medium inoculated with washed and starved diazotrophs under the conditions with no humin and reduced humin during 14 days of incubation. *Ensifer fredii* was incubated under molybdenum conditions, and *C. pasteurianum* under vanadium conditions, respectively. No organic carbon source was added to the culture medium. Controls without inoculation showed no increase in CO_2_ or ARA, regardless of the presence of the reduced humin. ND is not detected. The symbols “**” indicates significant differences at *p* < 0.01.

Fourteen heterotrophic diazotrophs from different taxonomic groups were used to test the effect of humin on BNF activity using the washed and starved cells in a modified nitrogen-deficient Ashby (MNDA) medium, based on the acetylene reduction activity (ARA) and total Kjeldahl nitrogen content. Two *Clostridium* spp. were included in 14 diazotrophs as the representatives of Clostridiales, which were major taxonomic group in the nitrogen-fixing anaerobic consortia promoted by humin in the previous study (Dey et al., 2021). The test was carried out by providing 10^9^ cells/ml of the diazotrophs initially (Supplementary Table S1). Figure 1 shows the effect of humin on ARA (indicator of nitrogenase activity) and total Kjeldahl nitrogen in the cultures of *A. vinelandii* and *C. pasteurianum* incubated in the absence of organic carbon source. Only the cultures with the reduced humin showed increases in both ARA and total Kjeldahl nitrogen. No increase was observed in the cultures with intact humin, oxidized humin, or no humin, in the absence of other organic carbon source. Other 12 heterotrophic diazotrophs also showed the increases in ARA (Supplementary Figure S2) and Kjeldahl nitrogen (Supplementary Figure S3) with the reduced humin in absence of other organic carbon source. Table 2 summarizes the specific BNF (nitrogenase) activities, i.e., BNF activity per cell, of the heterotrophic diazotrophs with the reduced humin in the absence of organic carbon source. Since the fixed nitrogen, detected as the increased Kjeldahl nitrogen, and promotion of ARA by the reduced humin showed a significant positive correlation (*r* = 0.779, *p* < 0.01) (Supplementary Figure S4), the ARA assay was used for evaluating the N-fixing activity in the subsequent experiments. The MNDA medium contained molybdenum; therefore, molybdenum nitrogenase activity was promoted by the reduced humin. The promotion was also observed in vanadium nitrogenase using *A. vinelandii* and *C. pasteurianum*, when vanadium was used in the incubation medium instead of molybdenum (Figure 2). Since BNF generally accompanies H_2_ production, this substance was also identified. Most diazotrophs produced slightly more H_2_ than that calculated from the stoichiometry of the BNF reaction with the reduced humin (Supplementary Figure S5, Supplementary Table S1). The role of humin as an electron mediator to promote BNF, and not as a source of organic carbon for diazotrophs, was confirmed by no increase in the CO_2_ production and the presence of ARA with the reduced humin in organic carbon source-free MNDA media (Figure 3, Supplementary Figure S6). The cultures with the reduced humin did not show an increase in the amount of CO_2_ but ARA (ethylene production). While, neither CO_2_ nor ethylene production was detected in cultures incubated without humin. These results indicate that the reduced humin promoted BNF activity of a broad spectrum of diazotrophs, including alpha-, beta-, gamma-, and delta-Proteobacteria, Firmicutes, Actinobacteria, and Archaea, which harbor nitrogenases classified into clusters I, II, and III, regardless of whether molybdenum or vanadium, without producing CO_2_. It was also confirmed that no other electroactive microorganism was involved in the BNF promotion of the individual heterotrophic diazotrophs with humin. That is, a broad taxonomic and enzymatic spectrum of diazotrophs can be activated using not genetically engineered, wild-type cells by extracellular electron transfer through humin.

**Table 2:** Effect of reduced humin on the N-fixation activity of various heterotrophic diazotrophs, as increased Kjeldahl nitrogen (Kjeldahl N) and acetylene reducing activity (ARA) under organic carbon source-free conditions.

**Taxonomic position**	**Name of the N-fixers**	***nifH* Cluster**	**Kjeldahl-N (10^**−18**^ mol-N/day/cell)**	**ARA (10^**−18**^ mol-C_**2**_H_**4**_/day/cell)**
Alpha Proteobacteria	*A. caulinodans*	I	62 ± 9	1.4 ± 0.09
	*E. fredii*	I	85 ± 3	3.1 ± 0.06
	*R. sphaeroides*	III	23 ± 10	1.0 ± 0.07
Beta Proteobacteria	*P. saccharophila*	I	43 ± 7	0.8 ± 0.05
	*D. gummosa*	I	53 ± 2	1.0 ± 0.04
	*R. gelatinosus*	III	31 ± 5	0.4 ± 0.02
Gamma Proteobacteria	*P. stutzeri*	I	60 ± 7	1.2 ± 0.08
	*A. vinelandii*	II	61 ± 11	1.2 ± 0.09
Delta Proteobacteria	*G. sulfurreducens*	III	30 ± 11	0.5 ± 0.06
Firmicutes	*P. macerans*	I	35 ± 4	0.6 ± 0.02
	*C. pasteurianum*	II	20 ± 4	0.2 ± 0.02
	*C. tyrobutyricum*	III	16 ± 4	0.2 ± 0.01
Actinobacteria	*N. cellulans*	I	23 ± 2	0.9 ± 0.09
Achaea	*M. barkeri*	III	35 ± 1	1.9 ± 0.17

### Acceleration of ARA by Reduced Humin Beyond the Level Achieved by a Carbon Source

**Figure 4:**
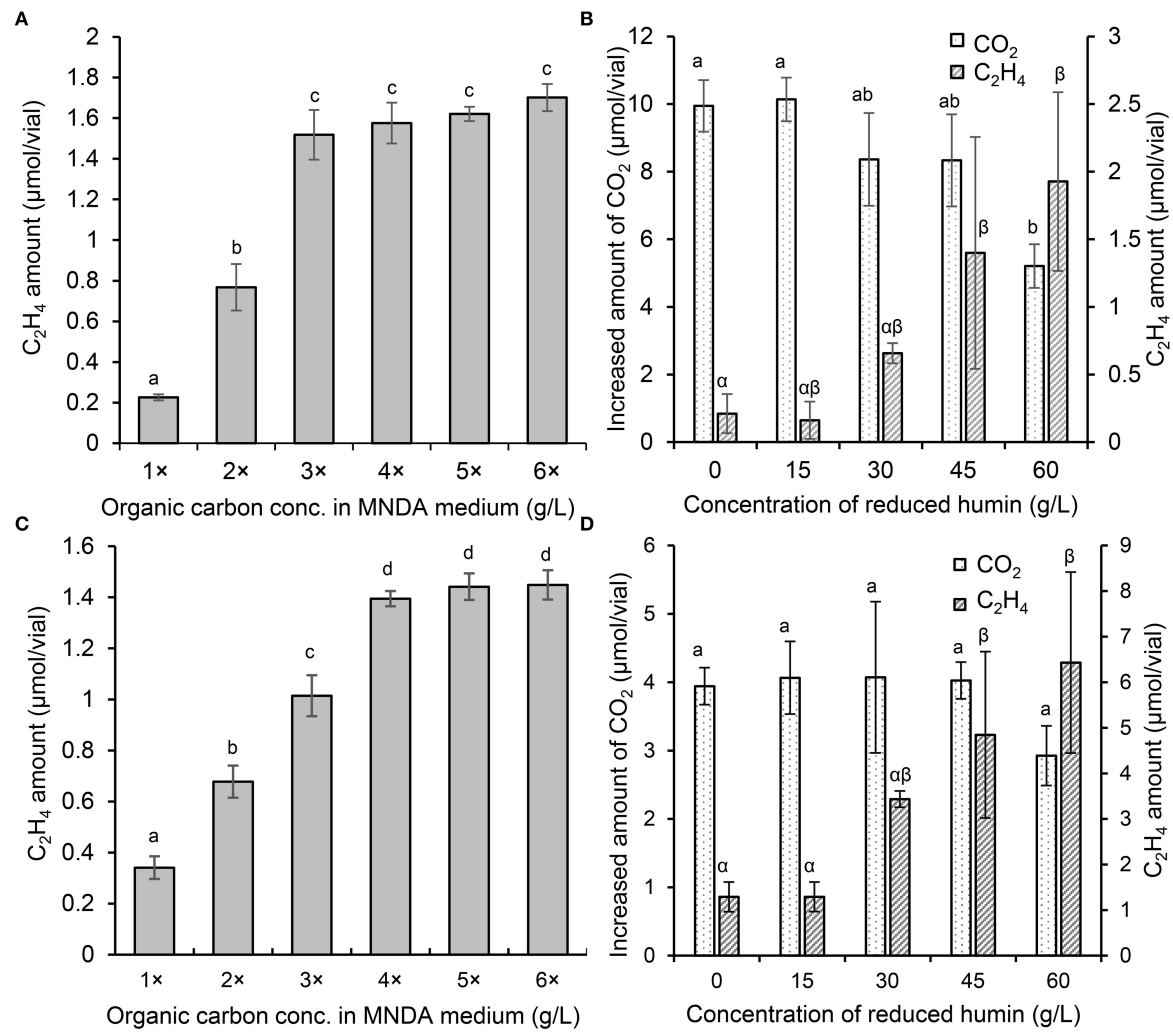
ARA and the increased amount of CO_2_ in the cultures of *E. fredii* and *C. pasteurianum* with different concentrations of organic carbon and the reduced humin. *Ensifer fredii* was incubated under molybdenum conditions, and *C. pasteurianum* under vanadium conditions, respectively. The ARA of *E. fredii* **(A)** and *C. pasteurianum* **(C)** in anaerobic medium with different concentrations of organic carbon. The ARA and the CO_2_ production in the cultures of *E. fredii* **(B)** and *C. pasteurianum* **(D)** with saturated organic carbon concentration and different concentrations of the reduced humin over seven days. No ARA was detected on day 0 or in the abiotic controls. Different letters indicate the significant difference at *p* < 0.05.

The ARA assays of washed and starved diazotrophs were performed with different concentrations of organic carbon sources to determine the saturated concentration of ARA. Figures 4A,C show that ARA increased to a maximum at four times of the organic carbon source in the culture of *C. pasteurianum* and at three times in *E. fredii*. There was no significant difference in ARA at higher concentration of the organic carbon sources than the saturated concentration. Obtained with the same trend, the saturated concentration of ARA was determined also for other diazotrophs (Supplementary Figure S7). Another ARA assay was performed to examine the effect of humin in a medium containing a saturated concentration of the carbon source. The results illustrated in Figures 4B,D show that the addition of the reduced humin further increased the ARA of *C. pasteurianum* and *E. fredii*, after ARA had previously reached a maximum with the organic carbon-saturated concentration. This further promotion was observed regardless of their taxonomic positions, *nifH* clusters, and nitrogenase type, as shown in Supplementary Figure S8. The promotion effect increased with an increase in the amount of the reduced humin, probably because of the greater number of supplied extracellular electrons. The promotion reached in the range between 194% of *A. vinelandii* (the lowest) and 916% (the highest) of *E. fredii* in the cultures with 60 g/L of reduced humin compared with the BNF activity of the diazotrophs tested with saturated organic carbon concentration but no humin, as 100%. The addition of the reduced humin did not increase the CO_2_ production, rather higher concentrations of the reduced humin inhibited the CO_2_ production (Figures 4B,D, Supplementary Figure S8B), which indicated the slackening of organic carbon metabolism. These results suggest that the supply of extracellular electrons through the reduced humin can further accelerate ARA and BNF activity.

### Promotion Mechanism of BNF Activity by Reduced Humin

**Figure 5:**
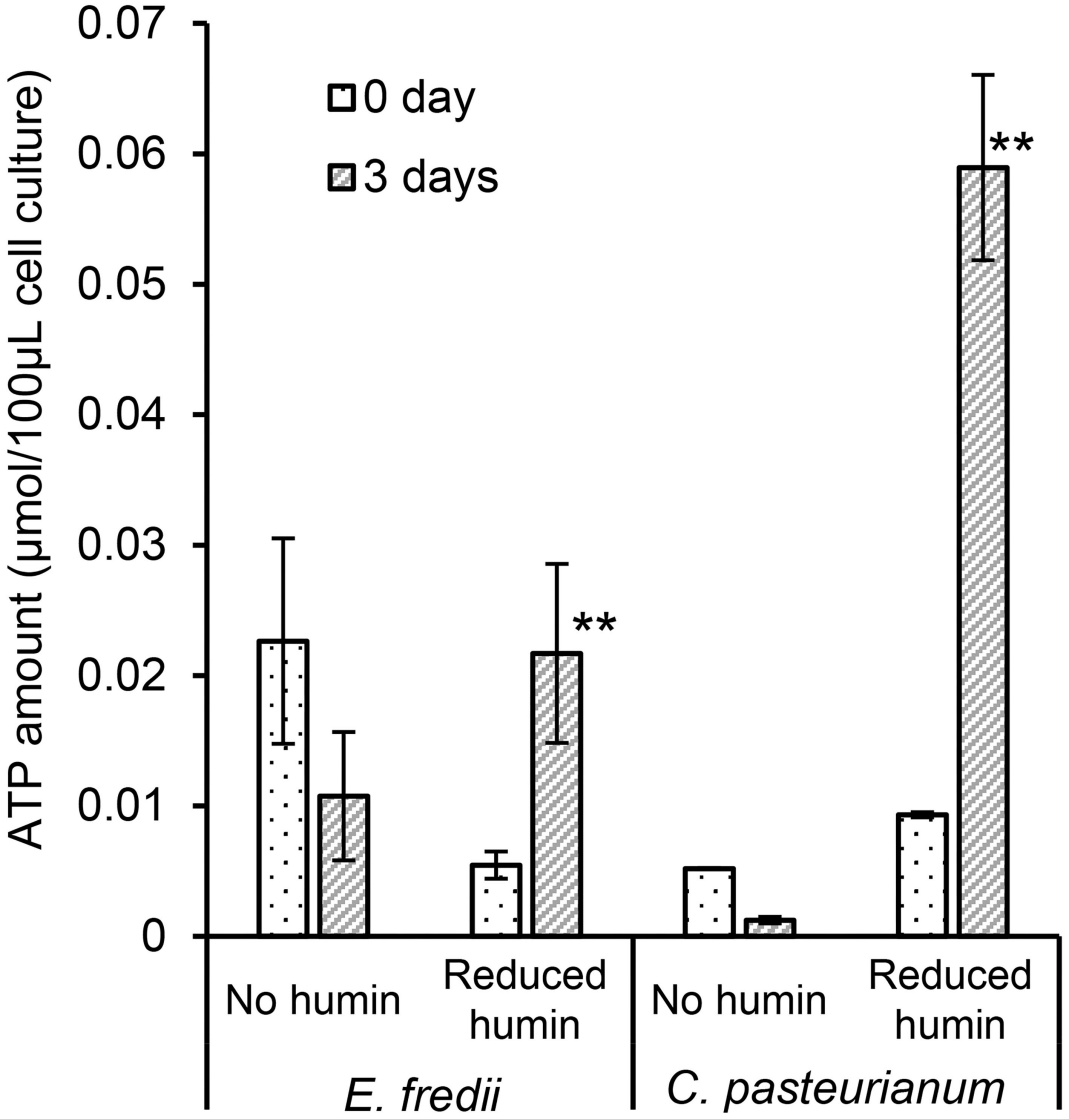
Effect of the reduced humin on the changes in ATP amount of washed and starved *E. fredii* and *C. pasteurianum* under the conditions with no humin and reduced humin during 3 days of incubation. *Ensifer fredii* was incubated under molybdenum conditions, and *C. pasteurianum* under vanadium conditions, respectively. No organic carbon source was added to the culture medium. Abiotic controls did not show any increase in ATP amount regardless of the presence of reduced humin (data not shown). Error bars represent the standard deviation of triplicate measurements. The symbols “*” and “**” indicate significant differences at *p* < 0.05, and *p* < 0.01, respectively.

To elucidate the BNF promotion mechanism of the reduced humin, ATP synthesis in the diazotrophs was examined under conditions of the reduced humin with no organic carbon source. The results (Figure 5, Supplementary Figure S9) showed that all six washed and starved diazotrophs harboring three different *nifH* clusters (clusters I, II, and III), and containing both molybdenum and vanadium nitrogenases, had increased ATP content in their cells without producing CO_2_ (Figure 3, Supplementary Figure S6). Controls without inoculation showed no increase in ATP levels, regardless of the presence of the reduced humin. This indicates that the reduced humin activated oxidative phosphorylation for ATP synthesis in broad taxonomic and nitrogenase spectra of diazotrophs. The extracellular electrons from the reduced humin most likely enter the diazotrophs through the cell membrane upstream of the oxidative phosphorylation process taking place.

**Figure 6:**
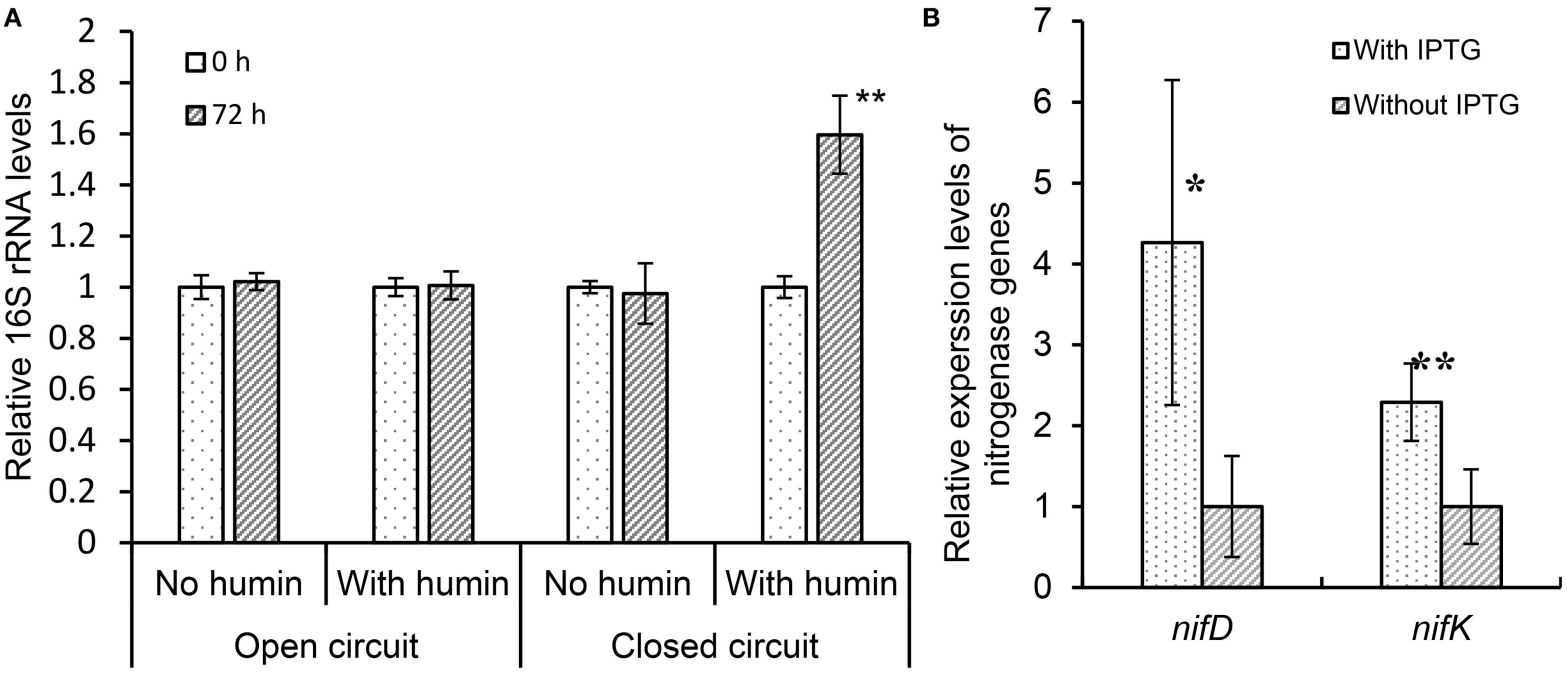
The growth of the *nifDK*-overexpression mutant (*nifDK*-OE) under nitrogen-deficient conditions in the presence of isopropyl β-D-1-thiogalactopyranoside (IPTG) **(A)** and the relative expression levels of nitrogenase genes *nifD* and *nifK* in the *nifDK*-OE mutant under the conditions with and without IPTG **(B)**. Error bars represent standard deviations of the four measurements. The symbols “*” and “**” indicate significant differences at *p* < 0.05, and *p* < 0.01, respectively.

Moreover, to examine whether humin can transfer electrons directly to nitrogenase protein without the involvement of nitrogenase reductase, the *nifDK* gene overexpression mutant (*nifDK*-OE) was cultured in an N-free M9 medium containing glucose under conditions of intact humin or no humin using an electrochemical system. The atmospheric nitrogen was provided as nitrogen source. The experiment was carried out by supplying electricity as an external electron source (closed circuit) and without the supply of electricity (open circuit) for both intact humin and no humin conditions (Supplementary Figure S1). The nitrogen fixation activity of the mutant was assessed indirectly by measuring the growth of the mutant using copy number of 16*S* rRNA gene before (0 h) and after 72 h of the incubation. Increase in the relative copy number of 16*S* rRNA gene, that is, the growth of mutant was observed only under the conditions of humin and a closed circuit; no growth was observed under the other conditions (the conditions of open circuit, and the condition of no humin and closed circuit), which was attributed to nitrogen deficiency of the mutant for growth (Figure 6A). The results indicate that the presence of humin aided the *nifDK*-OE mutant to receive electrons from external electron supply from the cathode, and these received electrons were utilized by MoFe protein (nitrogenase) of the mutant to fix N_2_ necessary for their growth. As a consequence, only the mutant under the condition of humin and closed circuit showed the growth. It is noted that, in natural BNF, MoFe protein receives electrons for nitrogen fixation through Fe protein (nitrogenase reductase), which was not present in the *nifDK*-OE mutant. In the *nifDK*-OE mutant, the expression of *nifD* and *nifK* was induced in presence of isopropyl β-D-1-thiogalactopyranoside (IPTG), which was added to all samples and confirmed by qRT-PCR (Figure 6B). These results suggest that humin is able to transfer electrons to nitrogenase (MoFe protein) directly because the mutant does not have nitrogenase reductase (Fe protein) genes. The mutant is also not an electroactive microorganism, indicating a direct electron transfer into the cell without relying on a biological mechanism.

## Discussion

Increases in ARA and total Kjeldahl nitrogen (Figure 1, Table 2, Supplementary Figures S2, S3) without an increase in the production of CO_2_ (Figure 3, Supplementary Figure S6) were observed in all the studied diazotrophs under the conditions with the reduced humin, but not under the conditions with intact, oxidized, and no humin. This suggests that the promotion of BNF activity of heterotrophic diazotrophs with humin is due to extracellular electron donation from the reduced humin, as no other electron donor was present in the culture. In the previous paper using the enriched diazotrophic consortia (Dey et al., 2021), the potential involvement of electroactive microorganisms in the consortia could not be ruled out for the extracellular electron transfer from humin to the diazotrophs. This study using isolates of diazotrophs confirmed no involvement of other electroactive microorganisms in the BNF promotion of the examined heterotrophic diazotrophs with humin. The stoichiometric production of H_2_ by diazotrophs with the reduced humin also supports the promotion of BNF through extracellular electron donation by humin (Supplementary Figure S5, Supplementary Table S1). Humin does not function as a carbon source for diazotrophs.

The diversity in the BNF promotion with humin was observed in the taxonomy of diazotrophs from Proteobacteria, Actinobacteria, and Firmicutes to Archaea, in the nitrogenases from clusters I, II, and III, and in both molybdenum and vanadium nitrogenases. Although the structures of cell membranes and cell walls differ among Proteobacteria, Actinobacteria, Firmicutes, and Archaea, BNF of all these diazotrophs were promoted by humin. Phylogenies of nitrogenase genes, *nifD and nifK*, generally agree with the nitrogenase clusters determined by *nifH* genes (Gaby and Buckley, 2011). In five major clusters, clusters IV and V are composed of *nifH* paralogues that are not involved in nitrogen fixation (Gaby and Buckley, 2011). The results that BNF activities of clusters I, II, and III were all promoted by the reduced humin, suggesting that all BNF with major nitrogenases, I, II and III would be promoted with the reduced humin. Alternative nitrogenases replacing molybdenum with vanadium were also promoted, as well as the molybdenum nitrogenase, although the structural difference has been suggested between the molybdenum and vanadium nitrogenases (Sippel and Einsle, 2017). These wide spectra of BNF promotion of heterotrophic diazotrophs with the reduced humin indicates a common mechanism of extracellular electron transfer from humin to diazotrophs, regardless of the differences in microbial cell structures and nitrogenase type. However, the common mechanism is not the function as reducing agent, since chemical reducing agents did not promote the BNF activity of consortia in the previous study (Dey et al., 2021).

The reduced humin increased further ARA of the diazotrophs that already reached the saturation of ARA with the organic carbon source. This further promotion of ARA with the reduced humin beyond the metabolic capacity of the diazotrophs ranged from 194 to 916% of the level achieved by the organic carbon source (Figure 4, Supplementary Figure S8). This shows the potential of the humin-assisted BNF promotion for technological applications. The highest promotion was observed in *E. fredii* (Figure 4B). Though humin can promote BNF activity of diverse heterotrophs, specific BNF activity of the diazotrophs using the reduced humin differed taxonomically. The specific BNF activity under the conditions with the reduced humin in organic carbon source-free anaerobic MNDA medium was higher in aerobic Proteobacteria (alpha, beta, and gamma, tested under anaerobic condition), especially in *E. fredii* (Table 2, Supplementary Table S1), while the low specific BNF activity was observed in anaerobic Firmicutes (*Clostridium* spp.). This taxonomic difference in specific BNF activity might be attributed to the difference in the structure of cell membranes and cell walls. It is well known that Firmicutes have a thick peptideglycan layer as cell wall, and Proteobacteria have cell membrane and outer membrane with a thin peptidoglycan layer between.

The extracellular electron donation from humin to diazotrophs are suggested in the promotion of BNF activities of diazotrophs with humin, since the promotion (ARA and Kjeldahl nitrogen) was only observed with the reduced humin but not with the other redox-states of humin (Figure 1, Supplementary Figures S2, S3). The extensive promotion of ARA of diazotrophs with the reduced humin, in which ARA was already saturated with the organic carbon, was observed without increase in the CO_2_ production (Figure 4, Supplementary Figures S7, S8). This suggested that the BNF promotion mechanism by the reduced humin was different from the one enhancing the metabolic activity upon the consumption of organic carbon.

Figure 5 and Supplementary Figure S9 show that the reduced humin resulted in the synthesis of ATP in diazotrophs. These results indicated that humin could donate extracellular electrons directly upstream in the cell membrane of the diazotrophs for ATP production by oxidative phosphorylation. In this ATP synthesis, soluble ions, such as Fe(III) and ${\text{HCO}}_{3}^{-}$, as well as insoluble humin itself present in the medium would be used as terminal electron accepters by the diazotrophs. Many microorganisms, including anaerobes, such as *Clostridium* sp. *Geobacter* sp., *Rhodobacte*r sp., aerobic microorganisms cultured under anaerobic condition, *Pseudomonas* sp., and *E. coli*, have been reported to use Fe(III) as terminal electron acceptor for ATP generation (Lovley, 1987, 1995). Moreover, *M. barkeri*, which is an archaeon used in this study, could use CO_2_ as terminal acceptor for energy generation. It has been reported that *M. barkeri* was able to use extracellular electrons to generate energy using CO_2_ as sole electron acceptor (Yee et al., 2019). In addition, solid-phase humin can work as a terminal electron acceptor for microbial respiration, as well as extracellular electron donor (Pham et al., 2021), just like water soluble humic substances (Lovley et al., 1996, 1999). Alternate functionality of solid-phase humin has been observed for the reactions with the same standard redox potential at −290 mV (*vs*. standard hydrogen electrode) as reported in the following: Anaerobic acetate oxidation by Geobacteriales with humin as the electron acceptor and the CO_2_ reduction to acetate by Clostridiales with humin as the electron donor (Laskar et al., 2019, 2020). It is plausible that heterogenic nature of solid-phase humin containing moieties with different redox levels (Pham et al., 2021, 2022) allow that one moiety functions as electron donor and another as electron acceptor. The increased production of ATP by the reduced humin would activate nitrogenase *via* nitrogenase reductase by transferring electrons from the reducing equivalents, which might be generated inside the washed and starved microbial cell under the reduced humin condition, to catalytic component of the nitrogenase enzyme. In addition, Figure 6 and Supplementary Figure S1 demonstrate the growth of *nifDK*-OE mutant only under the condition with humin and a close circuit, indicating the nitrogen fixation by the mutant. Since the *nifDK*-OE mutant did not have nitrogenase reductase, the results suggests that electricity was donated *via* humin to the nitrogenase present in the cytoplasm without relying on biological systems. Unlike other studies (Brown et al., 2016; Milton et al., 2016, 2017; Badalyan et al., 2019) on direct electron transfer to purified nitrogenase (MoFe protein), we have shown for the first time that extracellular electrons can be donated directly to nitrogenase (MoFe protein) present in cytosol in the cell *via* humin, for fixing atmospheric N_2_. However, humin is an insoluble organo–mineral complex with particle sizes larger than the microbial cells (Pham et al., 2021), and therefore could not carry electrons into the cell, like water-soluble redox shuttle (Kotloski and Gralnick, 2013). In the case of *M. barkeri*, the microbial electroactive system would enable the utilization of extracellular electrons in the reduced humin. However, other cases look like the direct transfer of extracellular electrons from humin into the microbial cells without depending on biochemical electron transfer system of the cell. Further studies should be warranted to elucidate the behind mechanisms in the extracellular electron transfer from solid-phase humin to the microbial cells and to develop technological applications.

## Conclusion

In this study, we have demonstrated that humin, a fraction of humic substances insoluble at any pH, as extracellular electron mediator, promoted nitrogen-fixing reaction of a broad spectrum of heterotrophic diazotrophs, including alpha-, beta-, gamma-, and delta-Proteobacteria, Firmicutes, Actinobacteria, and Archaea, which harbor nitrogenases classified into clusters I, II, and III, regardless of the nitrogenase type whether containing molybdenum or vanadium. The promotion level of the nitrogen-fixing ability of the diazotrophs exceeded beyond their capacity achieved by organic carbon source, suggesting potential of humin-promoted atmospheric nitrogen fixation as sustainable technology. In the promotion of nitrogen fixation, humin provided ATP production and a direct nitrogenase reduction by donating the extracellular electrons to the microbial cells. Although humin is an insoluble organo–mineral complex larger than the microbial cells, even a non-electroactive *E. coli* mutant utilized extracellular electrons from humin. These results suggest that the common mechanism present among the microorganisms, including the non-electroactive microorganisms in the extracellular electron, transfer *via* humin. The further research should be warranted to elucidate this electron transfer mechanism *via* humin.

## Data Availability Statement

The original contributions presented in the study are included in the article/Supplementary Material, further inquiries can be directed to the corresponding author.

## Author Contributions

TK and AK conceived the study and revised the manuscript. SD, TK, and AK designed the experiments. SD and TK performed the experiments and drafted the manuscript. All authors read and approved the final version of the manuscript.

## Funding

This research was financially supported in part by a Grant-in-Aid for Scientific Research (20H04363, 20K15431, 21K19862, JRP with NSFC FY2019) from the Japan Society for the Promotion of Science (JSPS) and by the Institute for Fermentation, Osaka (L-2019-3-003).

## Conflict of Interest

The authors declare that the research was conducted in the absence of any commercial or financial relationships that could be construed as a potential conflict of interest.

## Publisher's Note

All claims expressed in this article are solely those of the authors and do not necessarily represent those of their affiliated organizations, or those of the publisher, the editors and the reviewers. Any product that may be evaluated in this article, or claim that may be made by its manufacturer, is not guaranteed or endorsed by the publisher.
