# Supplementary material for: Promotion of Nitrogen Fixation of Diverse Heterotrophs by Solid-Phase Humin
Source: Front Microbiol. 2022 Aug 5;13:853411. doi: 10.3389/fmicb.2022.853411 (PMC9389315; doi:10.3389/fmicb.2022.853411)
Supplement: Supplementary file 1 [file Data_Sheet_1.pdf]

Supplementary Material

Promotion of nitrogen fixation of diverse heterotrophs by solid-phase humin

Sujan Dey [1, 2 †], Takuya Kasai [1,2, †], Arata Katayama[1,2*]

[1] Department of Civil and Environmental Engineering, Graduate School of Engineering, Nagoya University, Chikusa-ku, Nagoya 464-8603, Japan

[2] Institute of Materials and Systems for Sustainability, Nagoya University, Chikusa-ku, Nagoya, 464-8603, Japan

† These authors have contributed equally to this work and share first authorship.

*** Correspondence:**

Corresponding Author

katayama.arata@nagoya-u.jp

Number of Supplementary Tables: 2

Number of Supplementary Figures: 9

Supplementary Table S1: Cell numbers of different diazotrophs/mL in the inoculum, counted using fluorescence microscopy, in the test on the effect of humin on BNF activity

Daizotrophs	Cell number/ mL
Azorhizobium caulinodans JCM 20966	4.52×10^9
Ensifer fredii JCM 20967	3.84×10^9
Rhodobacter sphaeroides JCM 6121	4.88×10^9
Pelomonas saccharophila JCM 15912	4.62×10^9
Derxia gummosa JCM 20996	4.68×10^9
Rubrivivax gelatinosus JCM 21318	4.80×10^9
Azotobacter vinelandii JCM 21475	4.18×10^9
Pseudomonas stutzeri JCM 5965	5.54×10^9
Geobacter sulfurreducens DSMZ 12127	4.94×10^9
Paenibacillus macerans JCM 2500	9.24×10^9
Clostridium pasteurianum JCM 1408	7.17×10^9
Clostridium tyrobutyricum JCM 11008	1.20×10^9
Nocardia cellulans JCM 9965	9.80×10^9
Methanosarcina barkeri JCM 10043	5.14×10^9

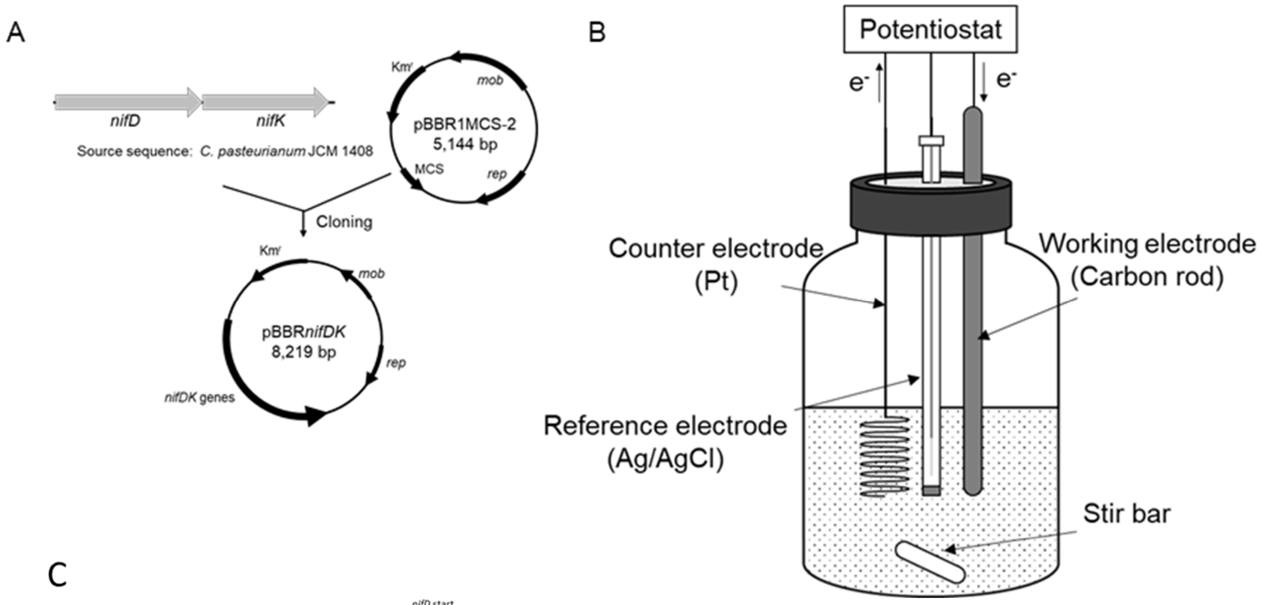

C

nifD start

CTATAAGATTTAATAAAAGTATTTAATTTTGATGAGGGGTGAATTTCGTGAGCGAAAATTTAAAAGACGAGATTTTAGAAAAATATATACCTAAAACTAAAAAGACTAGAAGTGGTCATATAGTTATAAAAACTGAAG

nifDK-F_KpnI

AAACACCAAATCCTGAAATAGTTGCTAACACAAGAACAGTGCCAGGAATAATCACAGCTAGAGGTTGTGCTTATGCAGGATGTAAAGGTGTTGTTATGGGACCAATAAAGGATATGGTTCACATCACACACGGACCT

qRT-nifD-F *qRT-nifD-R*

ATAGGATGTTCATTCTATACATGGGGTGGAAGAAGATTTAAGTCTAAACCAGAAAACGGTACTGGATTAAATTTTAATGAATATGTATTCTCTACTGATATGCAGGAAAGTGACATAGTTTTTGGTGGAGTTAATAAAT

TAAAAGATGCTATACATGAAGCATATGAAATGTTCCATCCAGCAGCTATAGGTGTTTATGCAACATGTCCAGTTGGTCTTATCGGTGATGATATACTAGCAGTTGCTGCAACAGCAAGCAAAGAAATTGGAATTCCAGT

TCATGCTTTTAGCTGTGAAGGTTATAAAGGTGTATCTCAATCTGCAGGTCACCATATAGCAAACAACACTGTTATGACTGATATCATAGGAAAAGGTAACAAGGAACAAAAGAAATATTCAATAAATGTCCTAGGAGA

GTACAACATTGGTGGAGATGCTTGGGAAATGGATAGAGTACTTGAAAAAATAGGTTACCATGTAAATGCAACTTTAACTGGTGATGCTACTTATGAAAAAGTACAAAATGCTGACAAAGCAGACTTAAACCTAGTTCA

ATGTCACAGATCTATCAATTATATTGCTGAAATGATGGAAACTAAATATGGTATTCCATGGATTAAATGTAACTTCATAGGTGTTGATGGAATAGTTGAAACACTTAGAGATATGGCTAAATGTTTTGATGATCCAGAA

CTTACTAAGAGAACTGAAGAAGTTATAGCAGAAGAAATAGCTGCGATTCAAGATGATTTAGACTATTTCAAAGAAAAACTTCAAGGAAAAACTGCTTGTCTATATGTTGGAGGATCAAGATCTCACACATACATGAAC

ATGCTTAAGAGCTTTGGTGTAGACAGTTTAGTAGCTGGATTCGAATTTGCTCACCGTGACGATTATGAAGGAAGAGAAGTTATACCAACTATCAAAATTGATGCGGACAGCAAAAACATTCCTGAAATAACTGTTACT

CCGGATGAACAGAAATACCGTGTTGTAATTCCAGAAGATAAAGTAGAAGAACTTAAAAAAGCAGGCGTTCCATTATCTTCTTATGGTGGAATGATGAAAGAAATGCATGATGGTACTATATTGATTGATGATATGAAT

CACCATGACATGGAAGTAGTACTAGAAAAGTTAAAACCAGATATGTTCTTTGCTGGAATAAAAGAAAAATTCGTTATCCAAAAAGGCGGAGTATTGTCAAAACAACTTCATTCATATGACTATAATGGACCATATGCA

nifD stop *nifK* start

GGTTTCAGAGGTGTAGTTAACTTTGGTCATGAACTTGTTAATGGTATCTATACACCAGCATGGAAGATGATAACACCACCTTGGAAAAAAGCTTCATCAGAAAGTAAAGTAGTTGTAGGAGGGGAAGCGTAATGTTA

GATGCAACACCAAAAGAAATAGTAGAAAGAAAAGCTTTAAGAATTAACCCAGCTAAAACTTGTCAACCAGTAGGAGCTATGTATGCTGCATTAGGTATTCATAACTGTCTCCCTCATAGTCATGGTTCACAAGGTTGTT

GTTCTTACCACAGAACAGTACTTTCAAGACACTTCAAAGAGCCAGCAATGGCTTCAACAAGTTCCTTTACTGAAGGTGCCAGCGTATTCGGTGGTGGTTCTAACATAAAGACAGCTGTTAAAAATATATTTTCATTATAC

AATCCTGATATAATAGCTGTTCACACAACTTGTTTATCAGAAACATTAGGTGATGATTTGCCTACTTATATCAGCCAAATGGAAGATGCAGGCAGCATACCAGAAGGTAAATTAGTTATCCATACAAATACACCAAGTT

ATGTTGGTTCACATGTTACTGGATTTGCTAATATGGTACAGGGTATTGTCAACTATTTATCTGAAAATACAGGTGCAAAAAATGGAAAAATAAATGTAATCCCTGGATTTGTTGGTCCAGCTGATATGAGAGAAATAAA

GAGATTATTTGAAGCTATGGATATTCCTTATATAATGTTCCCTGATACTAGTGGGGTTTTAGATGGTCCTACTACAGGCGAATACAAAATGTATCCAGAAGGTGGAACAAAGATTGAAGATTTAAAGGACACAGGAAA

TTCAGATCTTACTCTTTCTCTTGGAAGTTATGCTTCTGATCTAGGTGCAAAAACTTTAGAAAAGAAATGCAAAGTTCCATTTAAAACATTAAGAACTCCTATCGGTGTTTCAGCTACAGATGAATTCATAATGGCTTTATC

TGAAGCAACTGGTAAAGAAGTACCTGCTTCAATTGAAGAAGAAAGAGGTCAATTAATTGACTTGATGATAGATGCACAGCAGTATCTTCAAGGTAAAAAAGTTGCATTACTTGGAGATCCTGATGAAATTATTGCTCT

qRT-nifK-F

AAGCAAATTCATAATAGAATTAGGTGCTATACCAAAGTATGTGGTTACTGGTACTCCTGGTATGAAATTCCAAAAAGAAATCGATGCTATGCTTGCAGAAGCTGGTATAGAAGGAAGCAAAGTTAAAGTTGAAGGCG

qRT-nifK-R

ATTTCTTCGATGTACACCAATGGATAAAAAATGAAGGTGTTGATTTATTAATATCAAACACTTATGGAAAATTCATTGCAAGAGAAGAAAACATTCCTTTTGTTAGATTCGGATTCCCTATAATGGACAGATATGGACAT

TACTATAATCCAAAGGTTGGATACAAAGGTGCAATACGTCTAGTTGAAGAAATAACTAATGTTATCCTTGACAAGATTGAAAGAGAATGTACAGAAGAAGATTTTGAAGTAGTAAGATAATTTATCTTATAGTTATATA

nifK stop

TAAGTATTTTGGTGTCAATTCTATATAGTCATTCTATATGGAATTGACAATATTATATATTGATAAATG

nifDK-R_XbaI

Supplementary Figure S1 | Design of *nifDK* overexpression plasmid (A) and electrochemical reactor (B) and sequence of *nifDK* genes in *C. pasteurianum* JCM 1408 (C) .

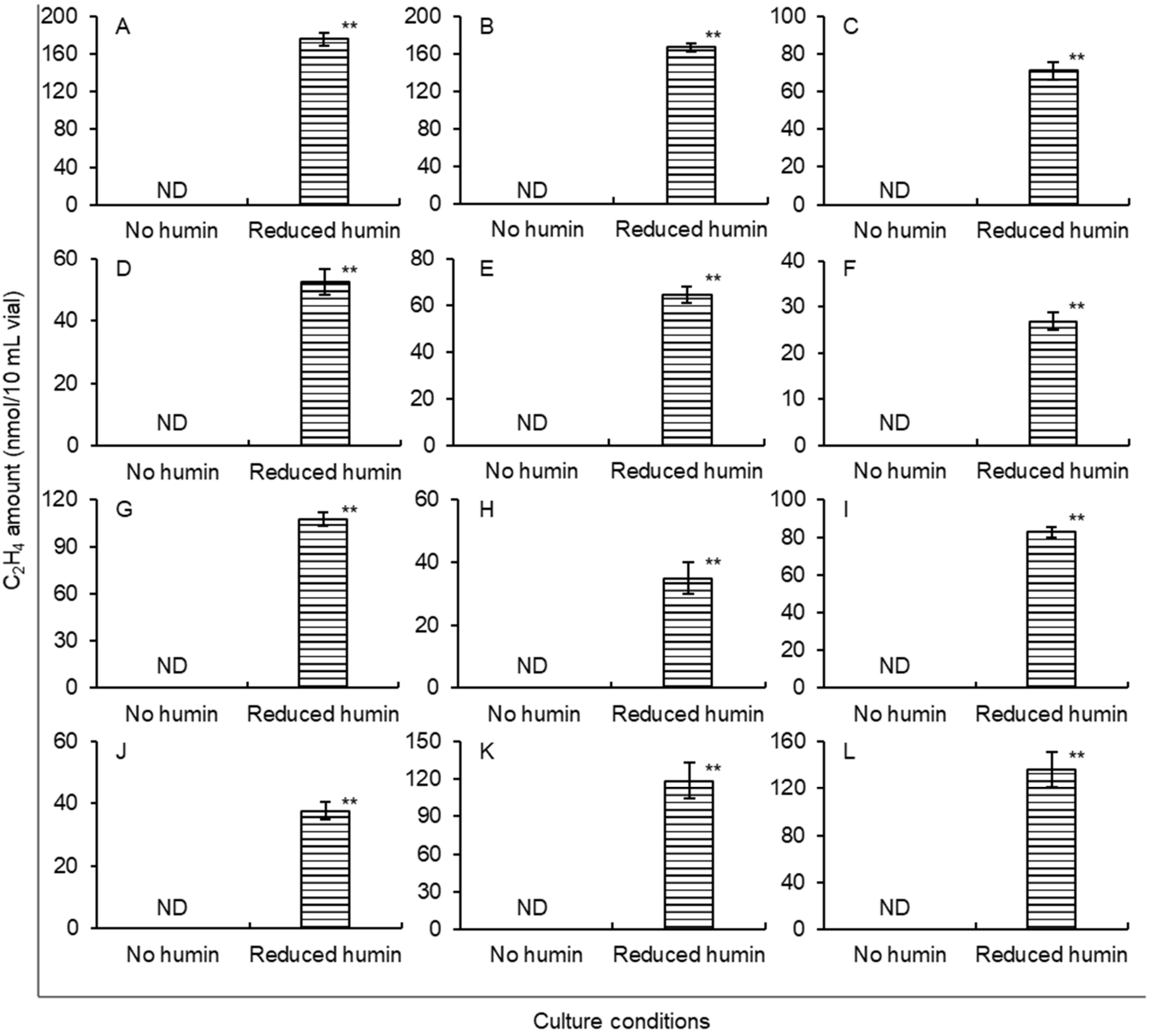

Supplementary Figure S2 | ARA of washed and starved diazotrophs with reduced humin. ARA is shown as the amount of ethylene produced in the cultures of washed and starved diazotrophs (*A. caulinodans*, *E. fredii*, *R. sphaeroides*, *P. saccharophila*, *D. gummosa*, *R. gelatinous*, *P. stutzeri*, *G. sulfurreducens*, *P. macerans*, *C. tyrobutyricum*, *N. cellulans*, and *M. barkeri*, labelled from A to L, respectively) with and without reduced humin. No organic carbon was added to the culture medium. ARA was detected only in cultures with reduced humin after seven days of incubation. The ARA assay was also performed using oxidized and intact humin cultures, but no C_2H_4 production was observed in all the diazotrophs (data not shown). No ARA was detected in any of the cultures on day 0. No ARA was observed in the controls without inoculation of the cultures, regardless of the presence of reduced humin (data not shown). ND represents not detected. Statistical analysis was performed using the t-test with the IBM SPSS ver. 21. Symbols ** indicates a significant difference at $p < 0.01$.

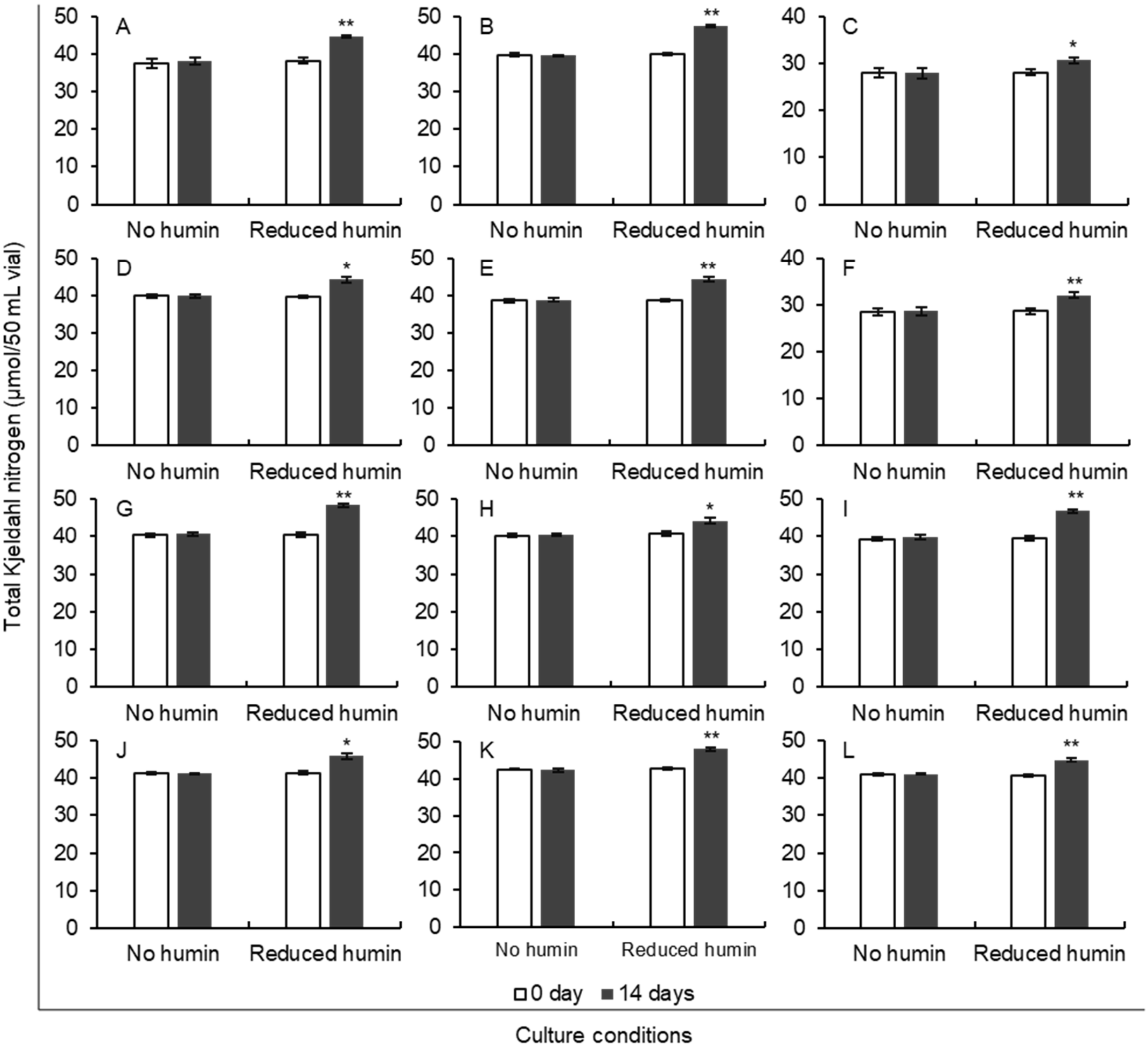

Supplementary Figure S3 | Change in total Kjeldahl nitrogen of the different washed and starved diazotrophs in the presence and absence of reduced humin. The nitrogen content of the diazotrophs is shown as the total Kjeldahl nitrogen in the cultures of washed and starved diazotrophs (*A. caulinodans*, *E. fredii*, *R. sphaeroides*, *P. saccharophila*, *D. gummosa*, *R. gelatinous*, *P. stutzeri*, *G. sulfurreducens*, *P. macerans*, *C. tyrobutyricum*, *N. cellulans*, and *M. barkeri*, labelled from A to L, respectively) with and without reduced humin. No organic carbon was added to the culture medium. Nitrogen content increased only in cultures with reduced humin after 14 days of incubation, whereas no increase was observed in the cultures without humin. No increase in nitrogen content was observed in the controls without inoculation of the cultures, regardless of the presence of reduced humin (data not shown). Statistical analysis was performed using the t-test with the IBM SPSS ver. 21. Symbols * and ** show significant differences at $p < 0.05$, and $p < 0.01$, respectively.

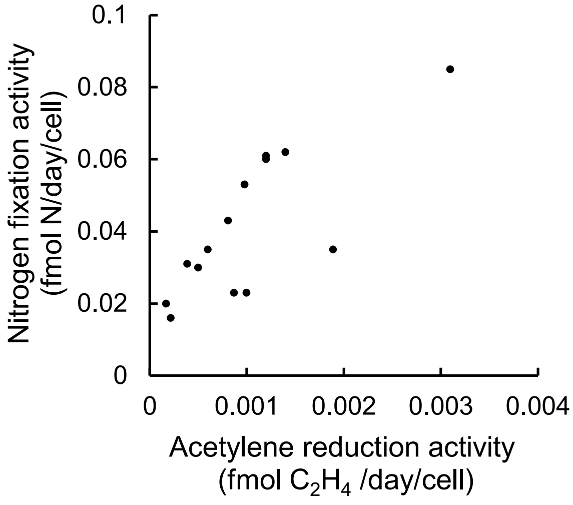

Supplementary Figure S4 | Correlation between nitrogen fixation capacity and ARA. Significant positive correlation (r = 0.779**) was observed between nitrogen fixation activity per cell (as the increase in total Kjeldahl nitrogen of the diazotrophs per cell) and ARA per cell in the cultures of all the studied diazotrophs. The cell numbers were counted directly using a fluorescence microscope after staining with DAPI. Pearson's correlation coefficient was calculated using the IBM SPSS ver. 21 (2-tailed, Symbol ** denotes $p < 0.01$).

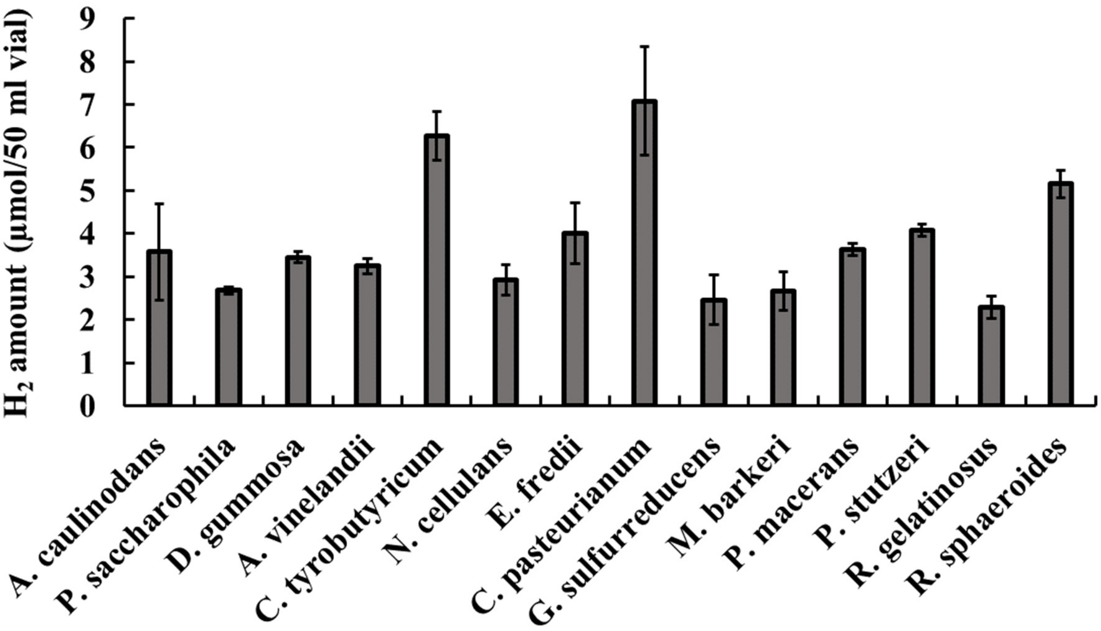

Supplementary Figure S5 | H_2 production by diazotrophs under reduced humin condition. No organic carbon source was added to the culture medium.

Supplementary Table S1 | Ratio of ammonia and hydrogen produced by washed and starved N-fixers under reduced humin condition

Name of the N-fixers	Mol ratio of NH_3 and H_2 (N:H)
Azorhizobium caulinodans	1.8 : 1
Ensifer fredii	1.9 : 1
Rhodobacter sphaeroides	0.50 : 1
Pelomonas saccharophila	1.7 : 1
Derxia gummosa	1.7 : 1
Rubrivivax gelatinosus	1.7 : 1
Azotobacter vinelandii	1.8 : 1
Pseudomonas stutzeri	2.0 : 1
Geobacater sulfurreducens	1.6 : 1
Paenibacillus macerans	2.0 : 1
Clostridium pasteurianum	0.48 : 1
Clostridium tyrobutyricum	0.71 : 1
Nocardia cellulans	1.8 : 1
Methanosarcina barkeri	1.6 : 1

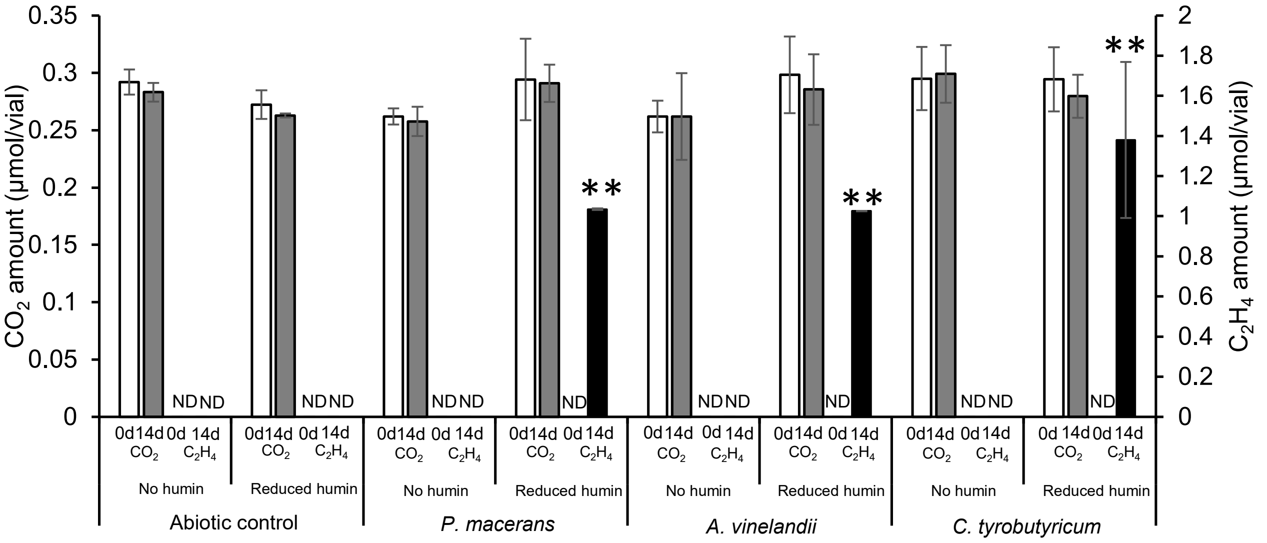

Supplementary Figure S6 | The CO_2 amount and ARA, as C_2H_4 amount, produced in the medium inoculated with washed and starved diazotrophs under the conditions with no humin and reduced humin during 14 days of incubation. No organic carbon source was added to the culture medium. Controls without inoculation showed no increase in CO_2 or ARA, regardless of the presence of reduced humin. All diazotrophs were incubated under molybdenum conditions, except for *A. vinelandii,* which was incubated under vanadium conditions. ND denotes not detected. The symbols ** indicates significant differences at $p < 0.01$.

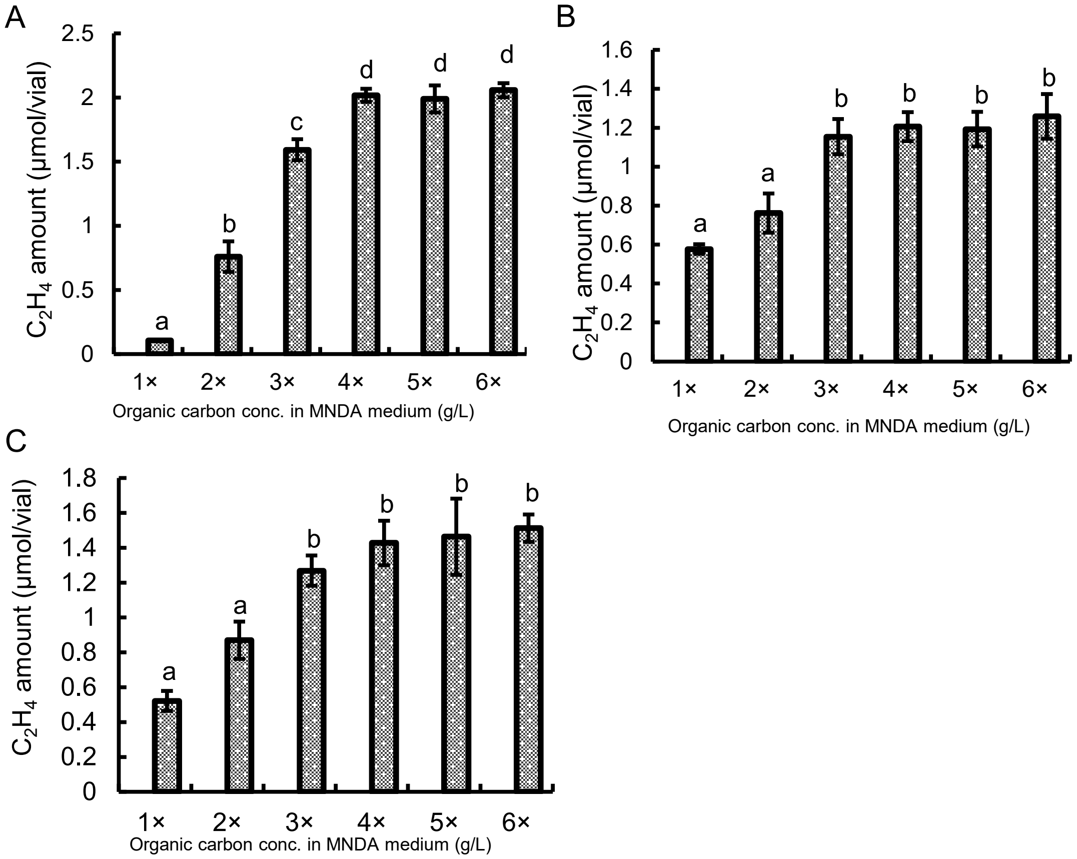

Supplementary Figure S7 |ARA, as the amount of ethylene produced, of washed and starved diazotrophs with different concentrations of organic carbon source. ARA of diazotrophs (*A. vinelandii***(A)**, *C. tyrobutyricum***(B)**, and *P. macerans***(C)**) in anaerobic medium with different concentrations of organic carbon. ARA increased with an increase in the concentration of organic carbon in the culture medium and reached a maximum at 4× (for *A. vinelandii* and *P. macerans*) or at 3× (for *C. tyrobutyricum* and *E. fredii*) concentrations of organic carbon, indicating the saturated concentration of organic carbon for maximum ARA. There was no significant difference in ARA from the 3×/4× to 6× concentration of organic carbon. ARA was not detected on day 0 for any of the conditions. Controls without inoculation showed no ARA on days 0 and 7. Symbol "×" represents the unit concentration of organic carbon in anaerobic MNDA medium, where 1× = 0.125 g glucose, 0.125 g soluble starch, 0.075 g Na-pyruvate /L for *A. vinelandii*, 1× = 1 g glucose and 0.125 g soluble starch /L for *C. tyrobutyricum*, 1× = 5 g mannitol/L for *E. fredii*, and 1× =1 g glucose/L for *P. macerans*, respectively. Statistical analysis was performed by one-way ANOVA followed by post-hoc analysis by Tukey's method using IBM SPSS ver. 21. Different letters indicate significant differences ($p < 0.05$). All diazotrophs were incubated under molybdenum conditions, except for *A. vinelandii*, which was incubated under vanadium conditions.

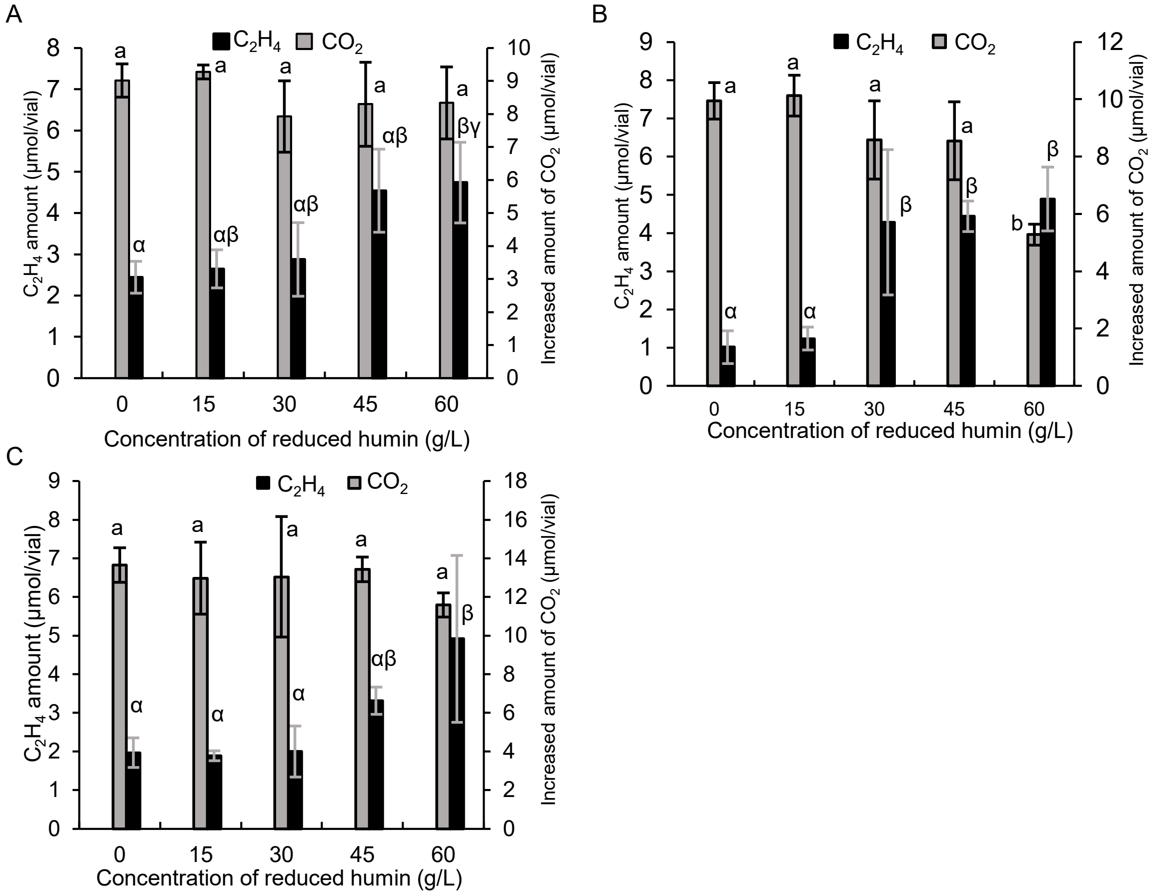

Supplementary Figure S8 | ARA, as the amount of ethylene produced, and the increased amount of CO_2, as the respiration rate, of washed and starved diazotrophs with saturated organic carbon concentration and different concentrations of reduced humin. ARA and the increased CO_2 amount in the cultures of the diazotrophs (*A. vinelandii*(**A**), *C. tyrobutyricum*(**B**), and *P. macerans*(**C**)) under the conditions with the saturated concentration (3×/4×) of carbon source and with different concentrations of reduced humin during seven days of incubation. ARA was not detected on day 0 for any of the conditions. Controls without inoculation showed no ARA on days 0 and 7. Symbol "×" represents the unit concentration of organic carbon in anaerobic MNDA medium, where 1× = 0.125 g glucose, 0.125 g soluble starch, 0.075 g Na-pyruvate /L for *A. vinelandii*, 1× = 1 g glucose and 0.125 g soluble starch /L for *C. tyrobutyricumi*, and 1× =1 g glucose/L for *P. macerans*, respectively. Statistical analysis was performed by one-way ANOVA followed by post-hoc analysis by Tukey's method using IBM SPSS ver. 21. Different letters indicate significant differences ($p < 0.05$). All diazotrophs were incubated under molybdenum conditions, except for *A. vinelandii*, which was incubated under vanadium conditions.

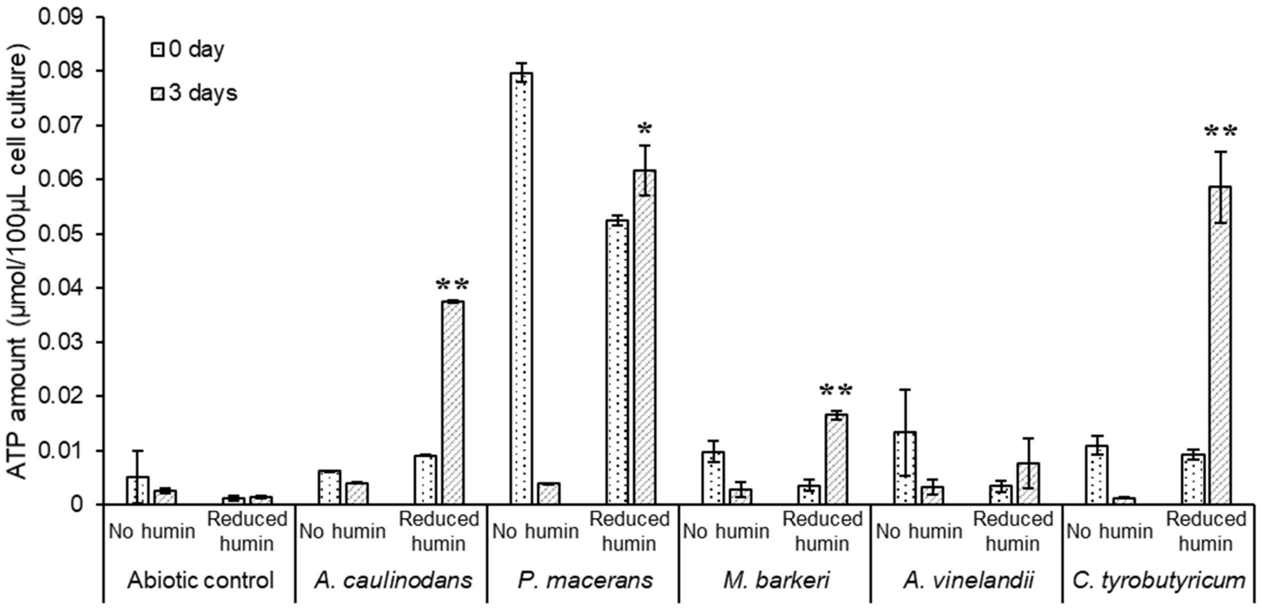

Supplementary Figure S9 | Effect of reduced humin on the changes in ATP amount of washed and starved diazotrophs under the conditions with no humin and reduced humin during three days of incubation. All diazotrophs were incubated under molybdenum conditions except *A. vinelandii* which was incubated under vanadium conditions. Error bars represent the standard deviation of triplicate measurements. Symbols * and ** show significant differences at $p < 0.05$, and $p < 0.01$, respectively。
